# Absolute measurement of fast and slow neuronal signals with fluorescence lifetime photometry at high temporal resolution

**DOI:** 10.1016/j.neuron.2025.08.013

**Published:** 2025-09-11

**Authors:** Bart Lodder, Tarun Kamath, Ecaterina Savenco, Berend Röring, Michelle Siegel, Julie A. Chouinard, Suk Joon Lee, Caroline Zagoren, Paul Rosen, Isa Hartman, Joshua Timmins, Roger Adan, Lin Tian, Bernardo L. Sabatini

**Affiliations:** 1 Department of Neurobiology, Howard Hughes Medical Institute, Harvard Medical School, Boston, MA 02115, USA; 2 UMC Brain Center, Department of Translational Neuroscience, University Medical Center Utrecht, Utrecht University, Utrecht, the Netherlands; 3 Max Planck Florida Institute for Neuroscience, Jupiter, FL, USA; 4 Department of Neuroscience and Physiology, The Sahlgrenska Academy at the University of Gothenburg, Göteborg, Sweden; 5 Altrecht Eating Disorders Rintveld, Zeist, the Netherlands; 6 Lead contact

## Abstract

Dynamic signaling by extracellular and intracellular molecules impacts downstream pathways in a cell-type-specific manner. Fluorescent reporters of such signals are typically optimized to detect fast, relative changes in concentration of target molecules. They are less well suited to detect slowly changing signals and rarely provide absolute measurements. Here, we developed fluorescence lifetime photometry at high temporal resolution (FLIPR), which utilizes frequency-domain analog processing to measure the absolute fluorescence lifetime of genetically encoded sensors at high speed but with long-term stability and picosecond precision. We applied FLIPR to investigate dopamine signaling in functionally distinct striatal subregions. We observed higher tonic dopamine levels in the tail of the striatum compared with the nucleus accumbens core and differential and dynamic responses in phasic and tonic dopamine to appetitive and aversive stimuli. Thus, FLIPR reports fast and slow timescale neuronal signaling in absolute units, revealing previously unappreciated spatial and temporal variation even in well-studied signaling systems.

## In brief

Fluorescence lifetime photometry at high temporal resolution (FLIPR) reports absolute fast and slow neuronal signals across time, brain regions, animals, and behavioral contexts. FLIPR, combined with the new dopamine sensor dLight3.8, allows for the simultaneous measurement of fast and slow changes in dopamine in freely moving mice.

## INTRODUCTION

The development of genetically encoded fluorescent indicators (GEFIs) for neurotransmitters, neuromodulators, and intracellular cascades has enabled the investigation of signaling in the brains of living animals.^1-10^ Most GEFIs are designed to change brightness upon substrate binding and thus are typically used with systems that measure fluorescence intensity over time.

Ideally, GEFIs would report signals in absolute, physiologically relevant units, such as numbers of action potentials or the concentration of a neuromodulator or second messenger. This is typically impractical because fluorescence intensity reflects both the state of individual sensors (typically bound or unbound to the molecule they detect) and bulk properties such as sensor concentration, which vary across cells, recording sites, and animals.^11,12^ For this reason, fluorescence intensity is usually converted into relative units by dividing by baseline intensity (dF/F), signal variance (Z scoring), or a ligand-insensitive signal such as the isosbestic point.^8,10,13^ These normalized signals can still be dynamically impacted by hemodynamic artifacts, animal movement, and differential photobleaching of signal and autofluorescence.^11,12,14-17^ Substrate-independent changes in fluorescence intensity also limit the timescale over which quantitative comparisons can be made—fluctuations in fluorescence intensity due to slow changes in substrate concentration are difficult to distinguish from bleaching or hemodynamic and motion artifacts.^10^ Photobleaching typically requires that relative measures be calculated over a rolling window, which hampers the detection of signals that occur more slowly than the window length.^10,13^ Consequently, standard fluorescence intensity measurements can, for instance, capture rapid transients in dopamine levels but not changes in slow, tonic dopamine.

An alternative approach is to monitor state-dependent changes in the fluorescence lifetime instead of intensity. Fluorescence lifetime is the average time after excitation that a fluorophore takes to release a photon and is an intrinsic property of each fluorescent molecule.^12^ As such, it is not dependent on sensor concentration or changes in intensity (as long as the sensor fluorescence is substantially brighter than autofluorescence).^9,17,18^ Therefore, lifetime measurements are insensitive to many of the caveats that limit absolute intensity imaging, including variability in expression, photobleaching, and hemodynamic and motion artifacts.^9,17,18^ Therefore, lifetime can be expressed in absolute units of time (typically picoseconds [ps] or nanosecond [ns]) and used to monitor signals across a wide range of timescales without post-processing.

The widespread use of fluorescence lifetime to monitor neuronal signals *in vivo* has been hampered by three main factors. First, lifetime is typically measured with time-correlated single-photon counting (TCSPC) systems that record the lifetime of one GEFI molecule at a time. Measurements are combined to form a histogram that is fit to calculate the population lifetime.^17,19,20^ Collecting sufficient individual emission events to achieve acceptable signal-to-noise is, due to hardware constraints, typically slow, limiting the temporal resolution to ~1 Hz.^17,19,20^ Second, existing lifetime detection systems are complex and expensive, often relying on highly specialized and fragile equipment.^9,10^ Third, most GEFIs do not exhibit state-dependent lifetime changes, and few fluorescence lifetime sensors of neuronal signals are available.^3,17,21-25^

With the goal of facilitating the high-speed absolute measurement of neuronal signals *in vivo* in absolute units and over a wide range of timescales, we designed a high-speed, precise, lowcost, robust, and easy-to-use fluorescence lifetime photometry (FLIP) system, termed FLIP at high temporal resolution (FLIPR). FLIPR relies on frequency-domain analog processing to calculate fluorescence lifetime at ps precision *in vivo*. We show that FLIPR enables accurate lifetime measurement in freely moving mice, preserving signals over wide temporal (DC to kHz) and fluorescence intensity ranges. We demonstrate the utility of FLIPR by using a fluorescence lifetime sensor to concurrently measure phasic and tonic dopamine in behaving mice. This reveals regional differences in tonic dopamine levels and changes in both absolute phasic and tonic dopamine during appetitive and aversive stimuli.

## RESULTS

### Design of FLIPR

**Figure 1.**
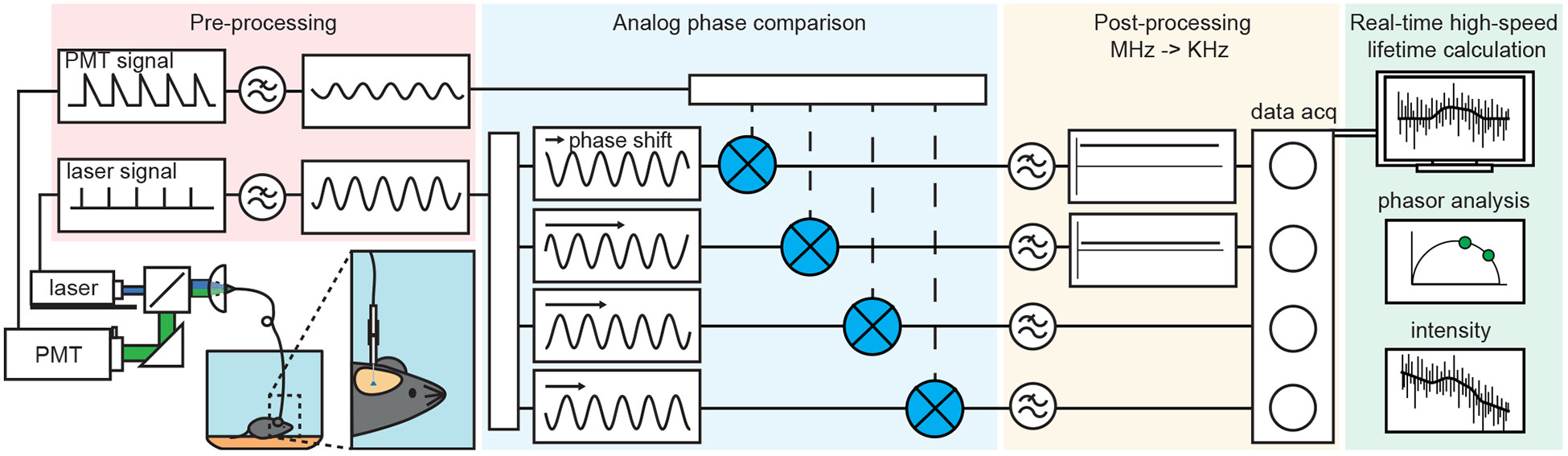
FLIPR system diagram FLIPR consists of an optical system, an analog processing unit, a DAQ system, and software allowing for real-time high-speed lifetime, phasor, and intensity measurement in freely moving mice. Excitation light produced by a 50 MHz pulsing 473 nm laser is directed into the brain of a mouse through an optical patch cord and fiber implant, where it excites a lifetime sensor. Emission light is captured by the same patch cord and delivered to a PMT. The laser pulse reference and PMT signals are low-pass filtered to capture the 50 MHz signal, amplitude modulated (preprocessing), compared in phase using frequency mixers (analog phase comparison), low-pass filtered to kHz levels and denoised (post-processing), and recorded by the DAQ system. Phasor and intensity signals are calculated and displayed in real time.

To enable high-speed and affordable fluorescence lifetime recordings in behaving animals, we developed FLIPR, which builds on previous work (Figures 1, S1, and S2).^8,9,19,26^ The excitation and emission paths are similar to those used in typical fiber photometry systems. A pulsed laser provides excitation light that is intensity modulated using an acousto-optic modulator and focused into a multimode optical fiber. The optical fiber couples end-to-end with a fiber stub implanted in the brain. Emitted fluorescence returns through the same path and, on exiting from the optical fiber, is collimated, separated from the excitation light, and directed to a photomultiplier tube (PMT).

FLIPR relies on a frequency-domain measurement approach, wherein the lifetime is calculated using an analog phase and intensity detection system (Figures 1, S1A, and S1B). The lifetime is calculated by comparing the phase difference of the PMT signal and the reference laser pulse. The electrical reference laser pulse signal is low-pass filtered (70 MHz corner) and referred to as the local oscillator voltage (VLO). The VLO is split into 4 channels that are shifted by 0, 0.5, 1, and 1.5 π radians, with the zero-phase shift being calibrated at 0 ns (or instantaneous lifetime) using a fluorescent lifetime standard. The PMT output is converted to voltage using a transimpedance amplifier, low-pass filtered at 70 megahertz (MHz), and separated into DC (VDC, the fluorescence intensity) and AC (VRF, radio frequency voltage) voltage components. The VRF is split into 4 copies, each of which is mixed with one of the phase-shifted reference signals. The mixers multiply each phase-shifted VLO (the laser reference) and the VRF (the PMT signal), producing outputs that depend on the relative phases and amplitudes of the two. The four signals (VIFs, intermediate frequency voltages) are passed through 1 kilohertz (kHz) low-pass filters and, along with the intensity VDC signal, recorded using a data acquisition (DAQ) system. The VIF and VDC allow for real-time calculation of lifetime and phasor components (see STAR Methods). Phasor analysis determines if the fluorescence follows a single exponential or multi-exponential decay: lifetimes with a single exponential decay have phasor components located on a semi-circle termed the universal circle (centered at g = 0.5, s = 0, and radius = 0.5), whereas lifetimes with a multi-exponential decay are located inside the universal circle.^27^ These signals are sampled by the DAQ system at the temporal resolution set by the dynamics of neuronal signaling (typically <1 kHz) as opposed to by the fluorescence lifetime (~10 gigahertz [GHz]).

The principle of operation of the FLIPR system is straightforward and previously described.^19^ However, we made several crucial changes to the analog computing unit to improve its accuracy and power stability as necessary for use in a real laboratory setting.

First, the amplifiers and frequency mixers produce nonlinear responses when the VRF approaches the 1 dB compression point.^28^ This produces a channel-specific reduction in voltage, which can cause substantial lifetime and phasor readout artifacts as a function of fluorescence intensity. To increase the stability of the lifetime readout across a broad intensity range, we used frequency mixers with a higher 1 dB compression point. In addition, we added RF amplifiers and attenuators to the VLO and VRF paths. We increased the VLO signal amplitude for optimal mixer performance, whereas we reduced the VRF signal amplitude to achieve a more linear response and reduce intensity-dependent voltage fluctuations. To accommodate the low VRF amplitudes, we selected a free-standing DAQ unit with high voltage accuracy and stability.

Second, the four phase shifters need to be set empirically using control voltages via analog control lines. However, the varying voltages necessary to produce 0–1.5 π radian shifts produce differential insertion losses. The resulting different VLO magnitudes across channels imbalance the mixer products, introducing inaccuracies in the lifetime estimation. To solve this problem, we accomplished most of the phase shift by altering the length of physical delay lines and used the phase shifters only to fine-tune the phase shift and calibrate the system. This minimizes differences between both phase shifters’ control voltages and VLO voltages.

Third, we observed electrical reflections in the system that affected mixer function. The mixers output the sum and differences of the VLO and RF frequencies, i.e., components at 0 and 100 MHz. Without the design features described below, the 100 MHz signal is reflected by a low-pass filter and re-enters the mixer, where it is multiplied with the VLO.^28,29^ This creates new 50 and 150 MHz signals that are again reflected at the low-pass filter. The interaction of these reflections with the VLO produces a low-frequency signal (VIF_reflection_) that sums with the initial low-frequency signal, introducing an artifact. Moreover, the amplitude of VIF_reflection_ depends upon the VLO phase shifts and therefore is channel-dependent. This imbalances the mixer outputs and perturbs the lifetime calculation. To solve this problem, we split the high-frequency and low-frequency mixer output using bias tees and pass the high-frequency signal through high dB absorbing attenuators and terminate them at 50 ohms, eliminating the reflection. Finally, we also reject higher frequencies produced by the phase shifters by placing low-pass filters before the mixers. These changes greatly increase the accuracy and stability of the phase detection system.

To control the FLIPR system, acquire data, and display fluorescence lifetime in real time, we developed MATLAB-based software, adapted from Sabatini lab electrophysiology acquisition software (Figure S1C). The cost of building the FLIPR system, excluding tools and a computer, is ~$38,000 (Table S1), with ~50% of the cost being the pulsed laser, which can, in theory, be shared across multiple systems. The total cost of building the previously published time-domain FLIP system is ~$68,000.

### *In vitro* validation

**Figure 2.**
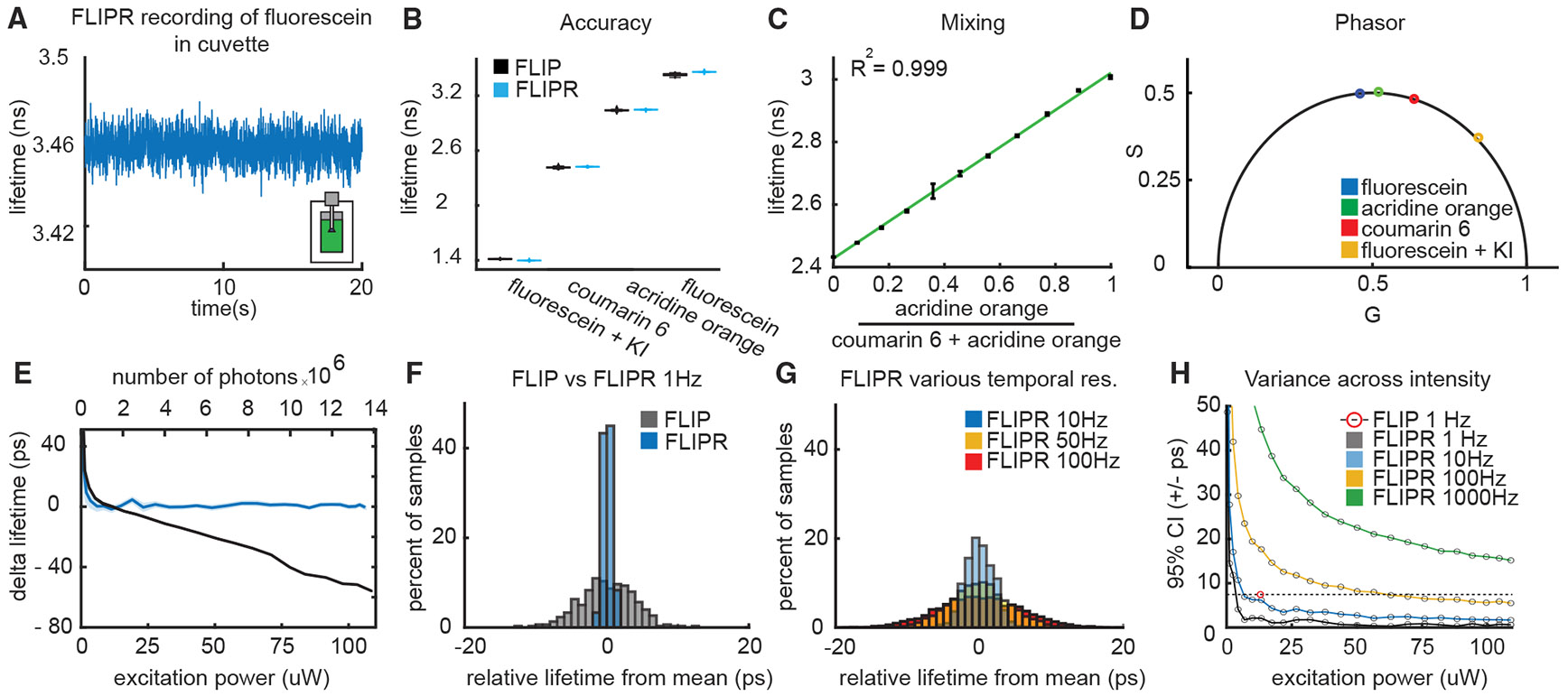
*In vitro* validation of FLIPR (A) Example fluorescence lifetime of fluorescein measured through a fiber optic placed in a cuvette with FLIPR at 100 Hz. (B) Comparison of mean lifetimes of coumarin 6, acridine orange, and fluorescein (in water or in water with 0.12 M potassium iodide) using FLIP (1 Hz) and FLIPR (10 Hz) measured as in (A). Boxplot elements: center mark, median; box limits, upper and lower quartiles; whiskers, furthest datapoints from the median. (C) Lifetimes of different ratios of acridine orange and coumarin 6 to determine linear accuracy of FLIPR. Green line: linear fit: R^2^ = 0.999. Error bars: SEM. (D) Phasor analysis of FLIPR measurements of coumarin 6, acridine orange, and fluorescein (data from B). Accuracy compared with the predicted point on the phasor plot was 99.59% SD = 0.28% for G and 99.57% SD = 0.30% S components. (E) Lifetime of coumarin 6 measured using FLIP and FLIPR with varying excitation power and number of detected photons (as reported by FLIP). As expected, due to photon pileup, FLIP underestimates the lifetime at higher photon counts. The lifetime measured using FLIPR remains stable across a broad range of fluorescence intensities. Traces show the means ± SEM across measurements. (F) Histograms showing variance of coumarin 6 lifetimes measured using FLIP at 1 Hz or FLIPR at 10 kHz followed by sample averaging and downsampling to 1 Hz. (G) Histograms showing variance of coumarin 6 lifetimes measured using FLIPR at 10 kHz and downsampled to 10, 50, and 100 Hz. (H) Variances of coumarin 6 lifetimes measured with FLIPR at different temporal resolutions and fluorescence intensities. The lifetime measured using FLIP at 1 Hz is shown for a power level at which this approach is accurate (red point and dashed line).

We compared the performance of FLIPR to that of the time-domain TCSPC-based FLIP system that we previously used for biological discovery^9,20,30^ (Figure 2). We built an optical layout to switch between lifetime measurements in time (FLIP) and frequency (FLIPR) domains (Figure S2). We used this approach to compare the lifetimes of fluorescent compounds coumarin 6 (7.1 × 10^−8^ M in ethanol), acridine orange (2.1 × 10^−7^ M in ethanol), and fluorescein (6.0 × 10^−6^ M in water or in water with 0.12 M potassium iodide) generated and collected with a fiber optic placed in a cuvette (Figure 2A). For all three fluorophores, the lifetimes were essentially identical whether estimated with FLIP or FLIPR (Figure 2B). To test the accuracy and linearity of FLIPR, the lifetimes of mixtures of acridine orange and coumarin 6 at varying ratios were measured. As expected for the lifetime of a mixture of two fluorophores with different individual lifetimes, the weighted ratios of the acridine orange/coumarin 6 intensity contribution and the measured lifetime were linear (Figure 2C, R^2^ = 0.999). Furthermore, phasor components of coumarin 6, acridine orange, and both fluorescein solutions fell on predicted positions on the universal half circle in the phasor plot (Figure 2D). These data indicate that lifetime measurements made with FLIPR are accurate and similar to those of conventional time-domain FLIP.

Most GEFIs were optimized to maximize state-dependent changes in fluorescence intensity. Furthermore, investigators may, depending on experimental needs, perform measurements at different excitation intensities, for example, to trade off signal-to-noise of rapid measurements vs. long-term stability. Thus, it is necessary to be able to perform accurate lifetime measurements over a wide range of fluorescence intensities without recalibration of the system.

To examine the stability and accuracy of lifetime measurements across intensities, the lifetime of coumarin 6 was measured at different photon rates (achieved through modulation of excitation power) using FLIP and FLIPR. As expected, lifetimes estimated with FLIP decreased at higher photon rates such that they were only accurate near the ~1 MHz photon detection rate, effectively limiting high-precision lifetime measurements to ~1 Hz. By contrast, FLIPR measurements were stable across the range of photon rates (0.5–14 MHz measured using FLIP) (Figure 2E), permitting lower variance measurements at 1 Hz sampling compared to FLIP (Figure 2F). Furthermore, we exploited the ability of FLIPR to operate at high speeds without deadtime to sample the fluorescence lifetime at 10 kHz, allowing us to set the final effective temporal resolution by downsampling after acquisition. This permits a user-defined trade-off between measurement variance and speed and allows temporal resolutions of 10–1,000 Hz while maintaining low variance (Figures 2G and 2H).

### *In vivo* validation

**Figure 3.**
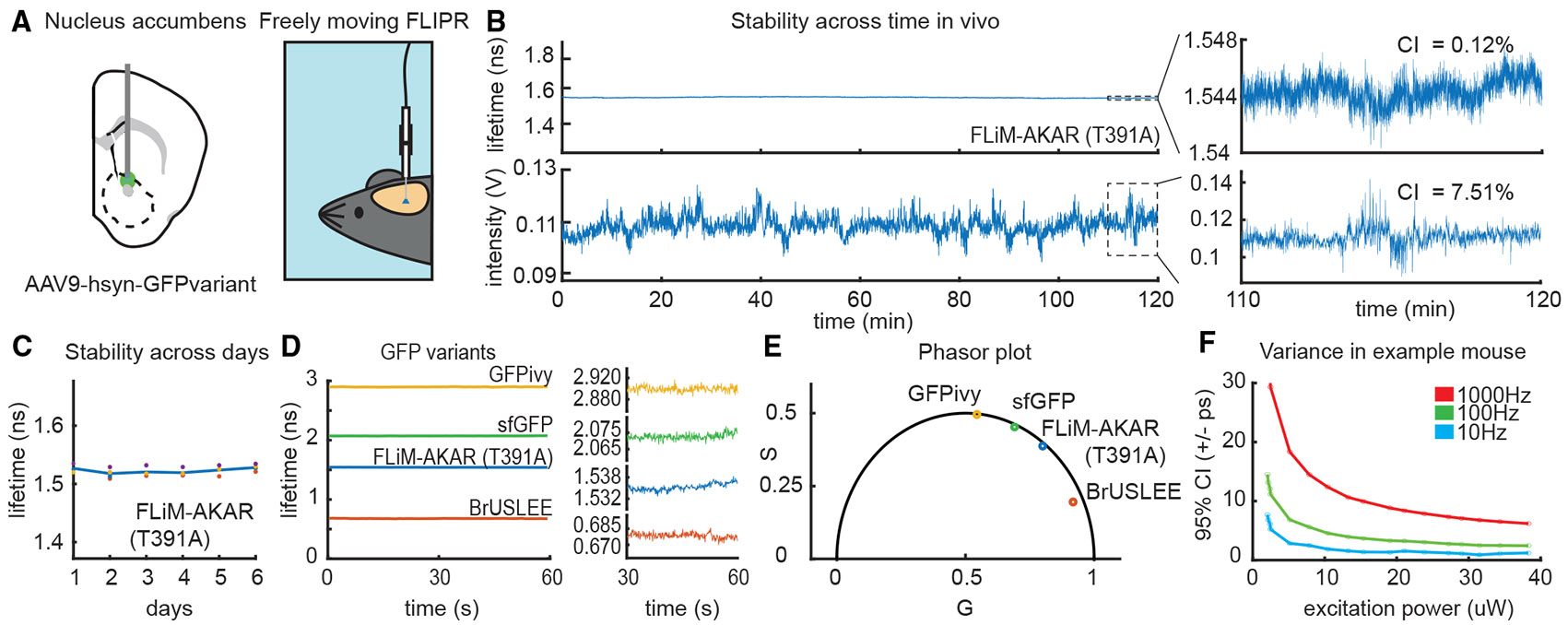
*In vivo* validation of FLIPR (A) Experimental setup for *in vivo* validation of the FLIPR system. The GFP variants GFPivy, superfolder GFP (sfGFP), FLiM-AKAR T391A, and GFP-BRuSLEE were individually expressed in the NAC, and the lifetimes were measured using FLIPR. (B) *In vivo* fluorescence lifetime and intensity of FliM-AKAR T391A, a pH-stable EGFP-based inactivated protein kinase A (PKA) sensor, were measured continuously for 2-h in a freely moving mouse. Lifetime was more stable than the intensity across the session (intensity CI = 7.75% from mean, lifetime CI = 0.35% from mean). Measurements are shown with the same range: mean ± 20%, downsampled and averaged to 1 Hz. Right, data from the last 10 min of recording on an expanded scale with corresponding variance, downsampled and averaged to 10 Hz. (C) Lifetime of FliM-AKAR T391A was measured in freely moving mice across 6 days. The lifetime did not change across days (*p* = 0.337, one-way repeated measures ANOVA, *n* = 4 hemispheres). (D) Lifetimes of GFPivy, sfGFP, FliM-AKAR T391A, and BRuSLEE were measured *in vivo* for 60 s in freely moving mice. Data are shown with all GFP variants on one axis (left) to show differences in absolute lifetimes and plotted individually (right) to show the stability of each measurement. (E) Phasor plot analysis of GFPivy, sfGFP, FliM-AKAR T391A, and BRuSLEE *in vivo* lifetime measurements. (F) Variance of FliM-AKAR T391A lifetime measured *in vivo* across excitation powers in an example mouse.

To examine the capabilities of FLIPR to perform continuous, prolonged measurements *in vivo*, we expressed FLiM-AKAR T391A, a pH-stable inactivated protein kinase A fluorescence lifetime sensor, in the nucleus accumbens core (NAC) and implanted a fiber optic above the injection site.^2^ After 4 weeks of expression, we measured the sensor lifetime daily for 6 days (Figures 3A-3C) while the mice explored an arena. The reported lifetime of FLiM-AKAR T391A remained stable across the 2-h session (Figure 3B, mean = 1.548 ns, confidence interval [CI] = 0.005 ns or 0.35% of the mean). By contrast, the intensity channel showed significant variance, possibly due to motion and hemodynamic artifacts (CI = 7.75% of the mean). However, these intensity changes minimally affected the lifetime, highlighting the insensitivity of FLIPR to artifacts typically found in intensity fiber photometry (R^2^ = 0.00011). Furthermore, there was no significant change in FLiM-AKAR T391A lifetime across days (Figure 3C, *p* = 0.223, one-way ANOVA). Similar stability was found for multiple GFP variants whose lifetimes vary over a wide range, such as GFP-BrUSLEE, FLiM-AKAR T391A, superfolder GFP (sfGFP), and GFPivy (Figures 3D and 3E; average lifetimes are 0.678, 1.537, 2.072, and 2.898 ns, respectively).^2,31-33^ Lastly, to demonstrate how users can trade off signal to noise (SNR) and long-term stability via selection of sampling frequency and excitation power, we measured the lifetime of FLiM-AKAR T391A *in vivo* at different excitation powers and calculated the variance of the signal at 10, 100, or 1,000 Hz sampling frequencies (Figure 3F). For most users, the excellent performance of ~5 ps CI at 100 Hz measurements at 10 microwatts (μW) excitation power will be adequate.

### dLight3.8 and FLIPR enable absolute dopamine measurements *in vivo*

**Figure 4.**
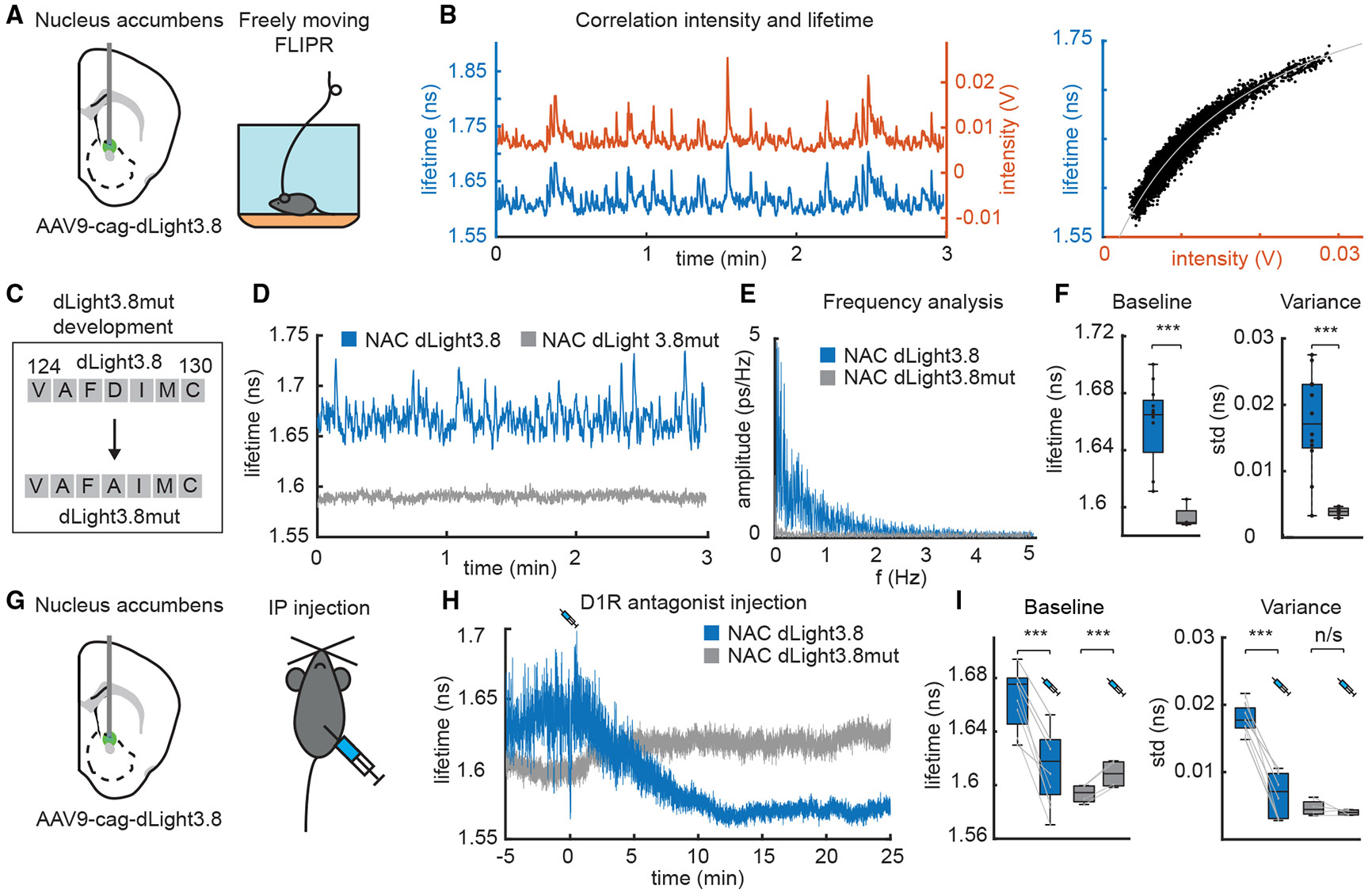
dLight3.8 is a lifetime reporter of dopamine (A) Schematic of experimental setup for *in vivo* FLIPR measurements of dLight3.8 fluorescence intensity and lifetime. The dopamine sensor dLight3.8 was expressed in the NAC. (B) dLight3.8 fluorescence intensity (red) and lifetime changes (blue) measured *in vivo* correlate (left). Right, the sublinear relationship between intensity and lifetime is expected and well-fit by theory (solid line). (C) A ligand-binding site mutant of dLight3.8 (dLight3.8mut) was generated by changing aspartate (D) 127 to alanine (A). Dlight3.8 and dLight3.8mut were expressed in the NAC for FLIPR analysis in freely moving mice. (D) Example traces of lifetime measurements of dLight3.8 (blue) and dLight3.8mut (gray). (E) Fourier transform frequency analysis of dLight3.8 and dLight3.8mut lifetimes. (F) Baseline lifetime (left) and variance (right) of dLight3.8mut (*n* = 4 hemispheres) are significantly reduced compared with those of dLight3.8 (*n* = 10) (two-sample t test, *p* = 0.0048 and 0.0034, respectively). Boxplot elements: center mark, median; box limits, upper and lower quartiles; whiskers, furthest datapoints from the median. (G) Schematic of the experiments used to measure the effects of D1R antagonism on dLight3.8 and dLight3.8mut lifetimes. Mice were injected i.p. with the D1R antagonist SCH23390 (10 mg/kg). (H) Example traces of *in vivo* lifetime measurements of dLight3.8 (blue) and dLight3.8mut (gray) following D1R antagonist injection (t = 0 min). (I) Average mean (left) and variance (right) of absolute lifetimes before and after D1R antagonist injection (lines connect data for individual mice). D1R antagonist injection significantly reduced the lifetime of dLight3.8 (paired sample t test, *p* = 2.76E–6, *n* = 8) and increased it for dLight3.8mut (paired sample t test, *p* = 0.0038, *n* = 4). In the same measurements, D1R antagonist decreased the variance of the lifetime of dLight3.8 (paired sample t test, *p* = 5.11E–8, *n* = 8) but did not affect that of dLight3.8mut (paired sample t test, *p* = 0.39, *n* = 4). Baseline values were calculated at t = −5 to 0 min and compared with t = 20–25 min. Boxplot elements: center mark, median; box limits, upper and lower quartiles; whiskers, furthest datapoints from the median.

We uncovered ligand-dependent fluorescence lifetime changes in the new dopamine sensor dLight3.8.^34^ To enable high-speed absolute dopamine measurements *in vivo*, we validated the use of dLight3.8 using FLIPR. First, we injected adeno-associated virus (AAV)9-cag-dLight3.8 into the NAC and placed a fiber above the injection site to measure fluorescence lifetime and intensity in freely moving mice (Figures 4A and 4B). Lifetime was strongly correlated with intensity (Figure 4B) and increased or decreased with optogenetic stimulation or inhibition, respectively, of midbrain dopamine neurons (Figure S3).^35^ The sublinear relationship between lifetime and intensity of dLight3.8 is expected as both the lifetime and brightness of the sensor change upon dopamine binding. This relationship is well explained by a model (Figure 4B) and does not reflect electronic artifacts (see STAR Methods). To further validate that the measured lifetime reflects dopamine concentration, we mutated the dopamine binding site of dLight3.8 (D127A) to generate dLight3.8mut (dLight3.8 D127A). dLight3.8mut had significantly reduced baseline lifetime mean and variance (Figures 4C-4F). Finally, intraperitoneal (i.p.) injection of SCH23390 (10 mg/kg), a non-competitive antagonist of type-1 dopamine receptors (D1R), on which dLight3.8 is based, reduced the baseline and variance of dLight3.8 lifetime significantly (Figures 4G-4I). Although D1R antagonist injection did not affect dLight3.8mut variance, it did slightly increase its lifetime. D1R antagonist application to brain slices slightly decreased dLight3.8mut lifetime, possibly reflecting allosteric effects of the D1R antagonist that alter dLight3.8mut lifetime in a context-dependent manner (Figure S4).

Lifetime measurements are in absolute units of time and, if performed accurately, can be compared across time, animals, and laboratories. In addition, if the *in vivo* properties of the indicator are known (such as the ligand concentration-dependence of lifetime and intensity changes), then the lifetime measurement can be used to calculate the fractions of sensor in the ligandbound and -unbound states as well as the ligand concentration (see STAR Methods). However, the properties of GEFIs, including their affinities, are often different *in vitro* and *in vivo*, and *in vivo* calibration is technically challenging. For these reasons, we chose to express the FLIPR measurements in absolute measures of time and did not attempt to convert them to dopamine concentration.

### *In vivo* measurement of absolute tonic and phasic dopamine signals with FLIPR

We examined the ability of measurements of dLight3.8 lifetime with FLIPR to report phasic dopamine signals, detect within-session tonic changes in dopamine, and compare basal levels of dopamine across manipulations and striatal regions. The striatum has functionally distinct regions: the NAC receives dopaminergic inputs from the ventral tegmental area that encode reward prediction errors, whereas the tail-of-striatum (TOS) receives dopaminergic inputs from the substantia nigra pars lateralis that encode threat and salience.^36,37^

**Figure 5.**
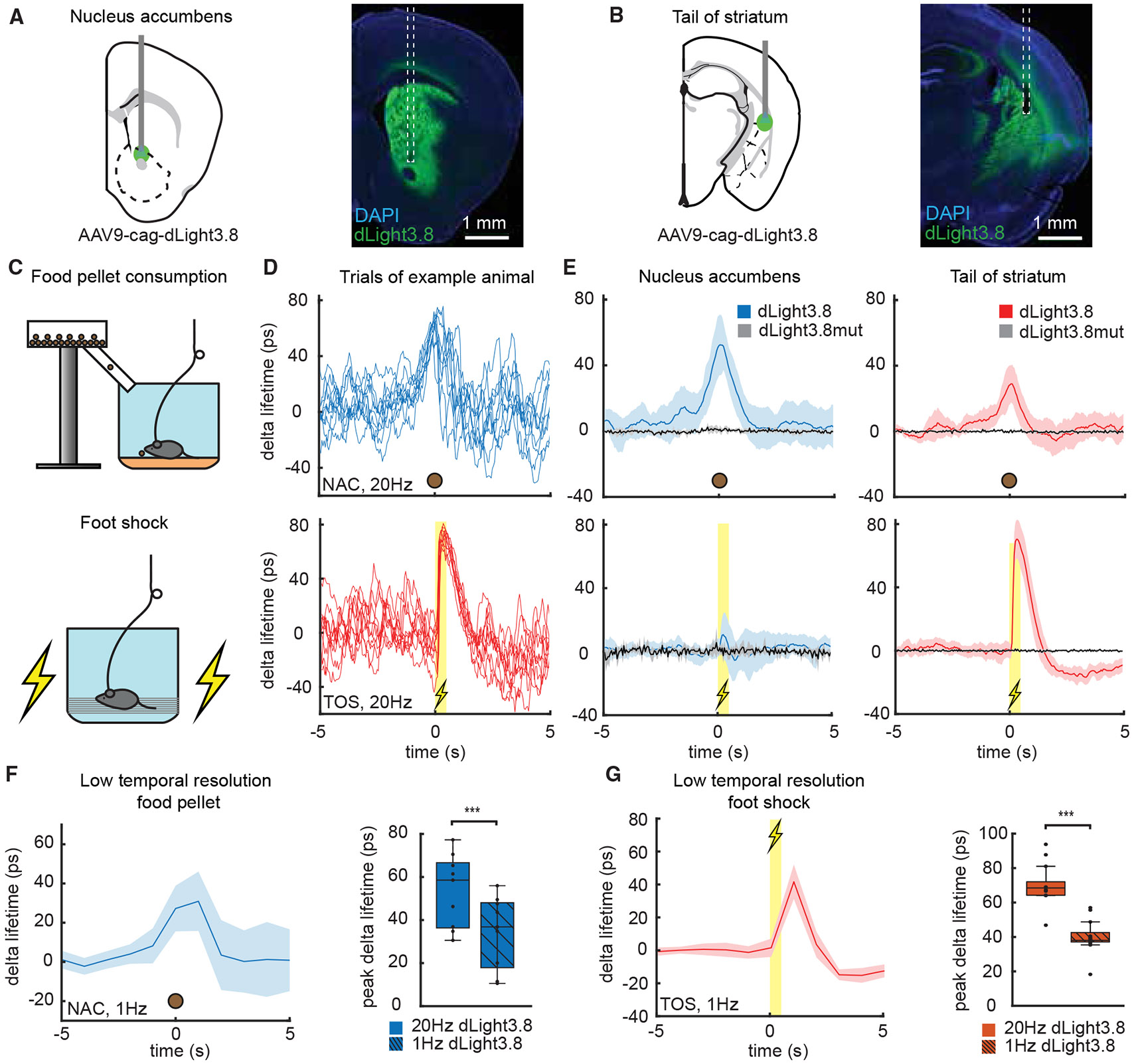
Phasic dopamine in the NAC and TOS during reward and punishment (A) Schematic of injection of AAV9-cag-dLight3.8 and fiber implantation in the NAC (left) and example histology of fiber placement (right). (B) Schematic of injection of AAV9-cag-dLight3.8 and fiber implantation in the TOS (left) and example histology of fiber placement (right). (C) Schematics of devices for spontaneous delivery of food pellets (top) or foot shocks (bottom). (D) Example lifetime measurements of dLight3.8 in individual trials (thin lines, t = 0 indicates the start of food pellet consumption) collected in NAC (blue, top) and TOS (red, bottom). (E) Average of lifetime transients of dLight3.8 and dLight3.8mut collected in the NAC (left) and TOS (right) evoked by food pellet consumption (top) (NAC: blue, *n* = 9 dLight3.8, gray *n* = 4 dLight3.8mut; TOS: red, *n* = 8, gray *n* = 4) or foot shocks (bottom) (NAC: blue, *n* = 12 dLight3.8, gray = 4 dLight3.8mut; TOS: red, *n* = 13 dLight3.8, gray = 6 dLight3.8mut). Traces show the means ± SEM across animals and hemispheres. (F) Average lifetime transient of dLight3.8 in the NAC in response to food pellet consumption, as in (E) (left, top), but downsampled to 1 Hz (left). NAC dLight3.8 peak delta lifetime was significantly reduced by downsampling during food pellet consumption (right, paired sample t test, *p* = 2.1365E–5, *n* = 9). Boxplot elements: center mark, median; box limits, upper and lower quartiles; whiskers, furthest datapoints from the median. (G) Average lifetime transient of dLight3.8 in the TOS in response to foot shock, as in (E) (right, bottom), but downsampled to 1 Hz (left). TOS dLight3.8 peak delta lifetime after foot shock was significantly reduced by downsampling (right, paired sample t test, *p* = 4.673E–13, *n* = 13). Boxplot elements: center mark, median; box limits, upper and lower quartiles; whiskers, furthest datapoints from the median excluding outliers.

We expressed dLight3.8 or dLight3.8mut in the TOS or NAC and placed a fiber optic above the injection site (Figures 5A and 5B). Mice were given food pellets and subject to foot shocks (Figures 5C-5E, S5A, and S5B). Pellet consumption evoked phasic increases in dopamine in the NAC and TOS, with those in the former being approximately 2-fold larger. Foot shocks minimally affected dopamine in the NAC but evoked large phasic transients followed by a small dip in the TOS. We compared absolute changes in phasic dopamine for NAC food pellet and TOS foot shock responses downsampled to 1 vs. 20 Hz to highlight the importance of the improved sampling rate of FLIPR to accurately estimate the amplitude of phasic transients (Figures 5F and 5G). Downsampling distorted the dLight3.8 waveform, underestimated the magnitude of the evoked change, and minimized the differences between signals in the TOS and NAC.

**Figure 6.**
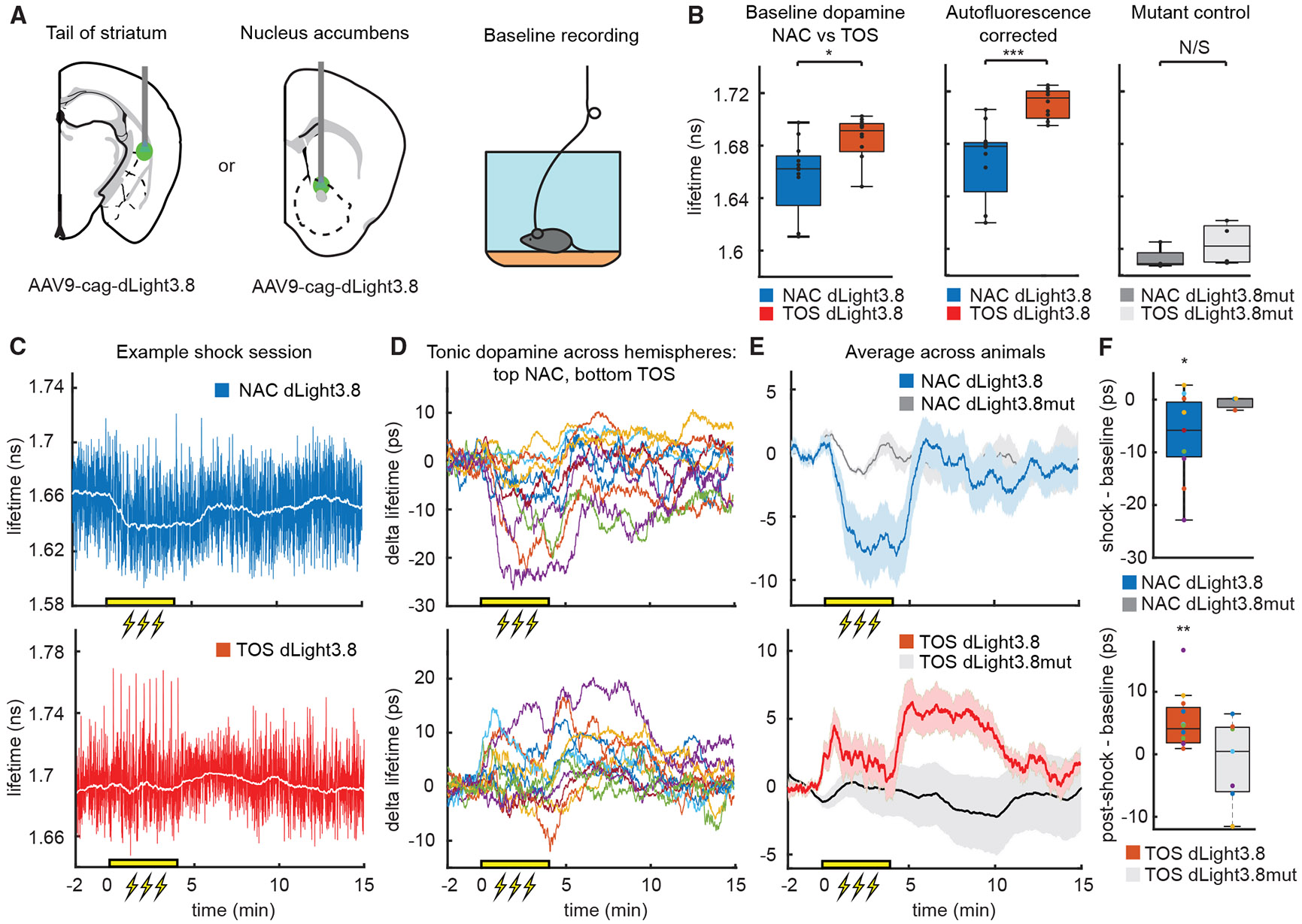
Regional and contextual variation in tonic dopamine measured with FLIPR (A) Comparison of tonic and phasic dopamine signals in NAC (right) and TOS (left) measured with dLight3.8 and FLIPR in freely moving mice. (B) Basal dLight3.8 lifetime is significantly elevated in the TOS (red, *n* = 12 hemispheres) compared with NAC (blue, *n* = 12) with (middle, two-sample t test, *p* = 3.533E–4) and without (left, *p* = 0.0219, two-sample t test) autofluorescence correction. There is no significant difference in dLight3.8mut lifetime between the TOS and NAC (right, *p* = 0.264, two-sample t test, NAC *n* = 4 and TOS *n* = 4 hemispheres). Boxplot elements: center mark, median; box limits, upper and lower quartiles; whiskers, furthest datapoints from the median. (C) Example session of dLight3.8 measured in the NAC (top) and TOS (bottom). Blue and red traces are downsampled to 10 Hz. The white line shows the median of the data calculated in a 1 min rolling window. The yellow bar indicates the period during which 10-foot shocks were delivered with random 20–30 s intervals. (D) As in (C), showing data for individual animals and measurement sites. During the foot shock period, tonic dopamine decreased in NAC (top) and increased in TOS (bottom). The signal increases further in TOS after the foot shock period. (E) As in (D), showing that, on average across mice, baseline lifetime of dLight3.8 decreased in NAC during foot shock (top, blue *n* = 11, gray *n* = 3) and increased in TOS after the foot shock period (bottom, red *n* = 11, gray *n* = 7). Traces: mean across animals. Yellow bar: foot shock period. Error bars, SEM. (F) dLight3.8 mean lifetime in the NAC significantly decreased during the shock period relative to baseline (prior 2 min) (top, *p* = 0.017, two-sample t test, blue dLight3.8 *n* = 11; *p* = 0.489, dark gray dLight3.8mut *n* = 3). dLight3.8 mean lifetime in the TOS significantly increased after shock trials relative to baseline (top, *p* = 0.002, two-sample t test, red dLight3.8 *n* = 11; *p* = 0.621, light gray dLight3.8mut *n* = 7). Boxplot elements: center mark, median; box limits, upper and lower quartiles; whiskers, furthest datapoints from the median.

To compare baseline dopamine in these distinct regions, we measured the lifetime of dLight3.8 in freely moving mice in a neutral chamber (Figure 6A). Tonic dopamine levels, as reported by dLight3.8 baseline lifetimes, were significantly higher in the TOS compared with the NAC (Figure 6B). To control for potential contributions of autofluorescence to the average lifetime, we investigated the level of autofluorescence *in vivo* in the TOS and NAC across days (Figure S5C). Autofluorescence is higher in the TOS and has a lifetime further from dLight3.8 than in the NAC. In both regions, autofluorescence is far lower than the dLight3.8 signal, such that autofluorescence correction maintained the difference in dLight3.8 lifetimes between TOS and NAC. Furthermore, dLight3.8mut lifetimes showed no significant differences between the NAC and TOS (Figure 6B), supporting the existence of true differences in tonic dopamine levels between the subregions.

Finally, to investigate dynamic changes in tonic dopamine across the session, we extracted basal dLight3.8 lifetime with a moving median filter (60 s) over the entire session of the previously collected foot shock data (Figure 6C). We calculated the delta lifetime through baseline (−2 to 0 s) subtraction. Although there were no foot shock-evoked phasic dopamine transients in NAC (Figure 5E), we observed a dynamic reduction in tonic dopamine during the 4-min period in which shocks were delivered. Tonic dopamine in NAC returned to the pre-shock baseline within ~1 min of the conclusion of the uncued shock period (Figures 6C-6F). By contrast, TOS tonic dopamine increased slightly during the shock period and increased for ~5 min after the shock period before returning to baseline (Figures 6C-6F). Similar changes in lifetime were not observed in NAC or TOS in mice expressing dLight3.8mut (Figure 6). Thus, FLIPR, used in conjunction with dLight3.8, can concurrently detect fast and slow changes in dopamine in the striatum, revealing regional specialization of tonic and phasic signaling evoked by behaviorally relevant events.

As neuromodulators often directly alter the activity or intracellular state of neurons, it is beneficial to measure signals from two sensors simultaneously. To examine if FLIPR and dLight3.8 can be used to concurrently measure dopamine and neuronal activity, we developed a two-color FLIPR system based on spectral separation of two excitation and emission paths (Figure S6A). We injected both dLight3.8 and Cre-dependent jRCaMP (a red calcium sensor with substrate-dependent lifetime changes) in the NAC of *Drd1-Cre* mice, which express Cre in D1R-expressing spiny projection neurons.^38^ The lifetimes of dLight3.8 and jRCaMP were measured simultaneously in freely moving mice (Figures S6B and S6C), and each showed different dynamics in response to food pellet consumption (Figures S6D and S6E).

## DISCUSSION

We designed and used FLIPR, a frequency-domain lifetime photometry system that enables high-speed fluorescence lifetime measurements in the brain of freely moving mice. FLIPR relies on an optimized analog-computing unit for high speed (1–1,000 Hz), high resolution, and accurate lifetime measurements. FLIPR is more affordable and robust than time-domain FLIP systems and is built from simple and low-cost analog components, making measurement of fluorescence lifetime *in vivo* accessible to the research community. We demonstrate the capabilities of FLIPR by exploiting the dopamine-dependent changes in the lifetime of the new dopamine sensor dLight3.8. FLIPR reveals heterogeneity in both phasic and tonic dopamine signaling across striatal regions and behavioral contexts. Moreover, we show that two-color FLIPR can be used to simultaneously measure levels of multiple molecules, such as dopamine and calcium. In short, FLIPR facilitates the adoption of robust lifetime photometry, allowing the community to take advantage of the new wave of lifetime reporters being generated.

### Comparison to other approaches for lifetime photometry

A key technical advantage that lowers the cost and complexity of FLIPR is that, because the computation is done in an analog processing unit, the sampling frequency of the digitizer can occur at the sampling frequency that the user desires, not at the high rates (~10 GHz) necessary to measure single photon arrival times. This is in contrast with most previous lifetime photometry systems, which rely on TCSPC systems that measure arrival times of individual photons relative to a sync pulse with ps resolution. Because of the complex electronics and timing mechanisms, these time-counting systems also typically have photon detection “dead times,” preventing counting of additional photons for ~100 ns after a photon is processed. This caps the theoretical maximal photon counting rates of the system. In practice, other technical concerns such as photon pileup reduce the maximal detection rate to ~1 MHz, which, when coupled with the need to collect several 100,000s of photons to provide an accurate lifetime measure, limits the effective temporal resolution to ~1 Hz.^9,17,39^ Photon rates beyond the limits imposed by electronics artificially lower the lifetime estimate, introducing signaldependent and dynamic measurement errors that can be difficult to detect and avoid *a priori*. There is no theoretical or technical limit on lifetime measurements at slow timescales (i.e., to monitor slow average changes), but due to limitations described above, it is not possible to measure fast and slow signals with the same system. State-of-the-art TCSPC boards relying on expensive equipment and hybrid PMT detectors have lower dead times and claim higher acceptable photon rates. However, these systems are still sensitive to measurement artifacts, such as the photon pileup effect. Moreover, the cost of such systems may be prohibitive for fiber photometry systems.

Frequency-domain lifetime measurement systems do not have photon detection dead times, and every photon that is detected by the PMT contributes to the calculation of the lifetime.^19^ This, and cost savings, motivated the development of the original analog processing unit for lifetime measurements.^19^ To achieve accurate, fast, and high dynamic range measurements with FLIPR, we suppressed electronic reflections, fixed phase delays, and properly power-balanced signals across the system. These improvements made FLIPR accurate across a large range of detected photon rates (0.5–14 MHz, measured by FLIP), allowing for lifetime measurements at 1–1,000 Hz with ps variance. Although properly used time-counting systems are the gold standard for lifetime measurements, we show that, at the same excitation power (and similar emission level), FLIPR has lower measurement variance, likely due to the rejection of photons by the electronics in time-domain systems and the ability to use high quantum efficiency PMTs.

The ability of FLIPR to accurately measure lifetime across a broad range of light levels permits its use for the analysis of fluorescence transients from sensors that have large conformationdependent intensity changes in addition to lifetime changes. As expected for accurate measurements of single-molecule properties, FLIPR measurements are insensitive to many artifacts and sources of variance that complicate comparison of photometry signals across time, sessions, and animals.^12^ FLIPR can be used with patch cords of different lengths and numerical apertures, as the calibration compensates for excitation and emission light traveling time. Finally, extracting lifetime from the analog outputs of FLIPR requires only simple matrix calculations.^19^ Phasor analysis allows for the easy identification of single- and multi-exponential lifetimes and calculation of the contributions of each fluorescent species in a multi-exponential sample. Altogether, FLIPR will be easy to implement for labs with fiber photometry experience, as surgical and behavioral requirements are identical for both techniques.

### Comparison to other approaches for absolute measurements of analytes *in vivo*

Fast scan cyclic voltammetry (FSCV) directly measures levels of compounds that can undergo ox/redox reactions at the surface of a carbon microfiber. FSCV is challenging to implement *in vivo* due to the complexity of data collection and analysis, caused by factors such as interference from other molecules and hemodynamic, electrical, and motion artifacts.^40-43^ Long-term chronic FSCV measurements are particularly difficult *in vivo* due to the degradation of the carbon fiber and reference electrodes, which complicates background subtraction and voltam-mogram identification.^44^ However, calibration of an FSCV probe *ex vivo* before or after implantation does allow for measurements of concentration.^40^ A recent preprint proposed a new method by which FSCV can be used in a ratiometric mode to derive relative phasic and tonic dopamine levels.^45^

FLIPR has several advantages over FSCV, such as its ease of adoption by laboratories, its ease of use in freely moving animals, the greater number of molecules and processes that can be measured, the ability to measure multiple molecules simultaneously, and the stability of absolute measurements across days using the same probe. FLIPR has the same surgical and behavioral requirements as fiber photometry and does not suffer from probe degradation, allowing for absolute measurement of molecules for the duration of sensor expression, typically for multiple months.

Although it allows for the capture of the absolute concentration of many molecules, microdialysis has a slow sampling rate (minutes) due to high sampling volumes, may not accurately report concentration due to rapid molecule degradation and depletion, causes acute damage during measurement, and is not compatible with real-time measurements of neuronal signals.^46^

### Tonic and phasic dopamine signaling

Tonic and phasic dopamine release determine the activation of dopamine receptors and downstream intracellular signals that govern neuronal plasticity and excitability.^47,48^ Most experimental efforts have focused on understanding phasic dopamine, with a few efforts examining tonic signaling.^10^ However, to truly understand dopamine function, it is crucial to measure phasic and tonic dopamine simultaneously, as the downstream impacts of phasic transients depend on baseline receptor occupancy, which is set by tonic dopamine levels.^30,49^ The interplay between tonic and phasic dopamine has been theorized to determine learning rates and contribute to the cause or treatment of several neuropsychiatric disorders, including Parkinson’s disease, drug addiction, schizophrenia, and attention-deficit hyperactivity disorder.^50-54^ The ability to measure both phasic and tonic dopamine simultaneously using FLIPR allows for in-depth analysis of the causes of these dysfunctions in disease models and for improved *in vivo* screening of candidate therapeutics.

We applied FLIPR to investigate tonic and phasic dopamine signaling in two regions of the striatum in response to appetitive and aversive stimuli. We observed increases in phasic dopamine during food pellet consumption in the NAC and foot shocks in the TOS, as expected based on the known functions of these regions, respectively, in reward and threat processing.^4,36,55,56^ We show that fast acquisition is necessary to accurately capture the timing and magnitude of phasic dopamine. Previous studies reported phasic dopamine transients in NAC in response to foot shock; however, we did not consistently observe such signals.^4,55^ Shock-evoked phasic dopamine transients are highly variable across the NAC, and our results do not exclude the existence of phasic dopamine foot shock responses in other regions.^55^

FLIPR reveals that, despite the lack of phasic dopamine responses to foot shock, NAC dopamine signaling is affected by these experiences: NAC basal dopamine decreases during the period that foot shocks are delivered. By contrast, tonic dopamine in the TOS increases after the shock period ends, confirming regional variation in tonic dopamine signaling. Such changes in baseline may facilitate or repress phasic dopamine transients and the intracellular signaling cascades that they activate. Therefore, decreased tonic NAC dopamine may alter reward value and reward-based learning during periods in which the animal detects threats. Similarly, the transient increase in tonic dopamine after the shock period in the TOS may alter threat sensitivity.

Highlighting the ability to compare across regions and animals, we also observed higher levels of tonic dopamine in the TOS compared with the NAC. This may reflect differences in the properties of dopamine axons in each region or the effects of local signals that impact dopamine release and clearance.

### Limitations and future

A limitation of lifetime photometry is the relative lack of fluorescent lifetime sensors. Lifetime changes are unexpected in sensors that allosterically couple biological processes to rearrangements of circularly permuted GFP (cpGFP). Nevertheless, lifetime changes have been observed in such sensors, including in dLight3.8 and others.^17^ Sensors designed for lifetime measurements will ideally have only small intensity changes, which facilitates accurate measurement of lifetimes in the dim state and increases the linearity of lifetime changes relative to the biological process of interest.

Although FLIPR provides an absolute measurement that can be compared across time, brain regions, and animals, it is not a calibrated measure. In theory, lifetime measurements can be calibrated to different concentrations of molecules in the same manner as FSCV; however, the lifetimes of sensors *in vitro* and *in vivo* may differ. Thus, translating absolute lifetime measurements into concentrations of analytes is difficult (see STAR Methods for one calibration approach). To measure absolute signals using FLIPR, we recommend careful comparison of sensor brightness to autofluorescence and the use of a ligand-binding mutant to control for ligand-independent effects on the sensor.

The FLIPR system described here was built by hand and is not difficult to reproduce. However, it does require attention to detail and may be challenging for some. Production of a printed circuit board that can be ordered with surface-mount electronics in place will simplify adoption. In addition, replacement of PMTs with silicon-based detectors and of the pulsed laser with other high-frequency (>20 MHz) modulated light sources may simplify power control and permit easily changing the excitation and emission spectra to accommodate new fluorophores. Despite these limitations, FLIPR allows for absolute fluorescence lifetime measurement at low cost, permitting fast and slow neuronal signals to be easily compared across timescales, animals, and conditions.

## STAR★METHODS

### EXPERIMENTAL MODEL AND STUDY PARTICIPANT DETAILS

Experimental manipulations were performed in accordance with protocols approved by the Harvard Standing Committee on Animal Care following guidelines described in the US National Institutes of Health Guide for the Care and Use of Laboratory Animals. *C57BL/6J* (000664) mice and *DAT-IRES-Cre* (B6.SJL-Slc6a3tm1.1(cre)Bkmn/J, 006660) were acquired from the Jackson Laboratory.^56^ *Drd1a-cre* (B6.FVB(Cg)-Tg(Drd1-cre)EY262Gsat/Mmucd, 030989-UCD) mice were acquired from MMRRC UC Davis.^58^ Mouse ages were typically 2-4 months, and male and female mice were used in approximately equal proportion. Mice were housed on a 12hr dark/12hr light reversed cycle. Mice were group-housed unless food restricted. As measurements performed here have never been performed before, no power-based sample size calculation could be performed.

### METHOD DETAILS

#### Viruses

Recombinant adeno-associated viruses (AAVs) of serotype 9 or DJ/9 were used to express transgenes of interest in either Cre-recombinase dependent or independent manner. AAVs were packaged by commercial vector core facilities (Janelia Vector Core and UNC Vector Core) and stored at −80°C upon arrival. Viruses were used at a working concentration of 10^12^ to 10^14^ genomic copies per ml. 250-300 nl of virus was used for all experiments. The following viral plasmids are available on Addgene: AAV-FLEX-FLIM-AKART391A (#60446), AAV1-syn-FLEX.NES-jRCaMP1b (#100850) and AAVDJ9-nEF-Con/Foff 2.0-ChRmine-oScarlet (#137161). AAV-hsyn-dLight3.8mut, AAV-hsyn-sfGFP, AAV-hsyn-BrUSLEE and AAV-hsyn-Ivy are available upon request to Dr. Bernardo Sabatini. AAV-cag-dLight3.8 is available at UNC neurotools.

#### Surgery

Mice were given *ad libitum* oral carprofen one day before surgery. During the stereotactic surgery, inhaled isoflurane was used as anesthesia. Surgeries were performed using a stereotaxic frame (David Kopf Instruments) and 250-300 nl of virus was injected stereotactically at the following titers: AAV9-cag-dLight3.8 1 x 10^13^gc/ml, AAV9-hsyn-dLight3.8mut 2.1 x 10^13^ gc/ml, AAV9-hsyn-sfGFP 1.4 x 10^12^ gc/ml, AAV9-hsyn-BrUSLEE 5.4 x 10^12^ gc/ml, AAV9-hsyn-Ivy 7.8 x 10^12^ gc/ml, AAV-FLEX-FLIM-AKART391A 7.6 x10^12^, AAV1-syn-FLEX.NES-jRCaMP1b 9 x 10^12^ gc/ml, AAVDJ9-nEF-Con/Foff 2.0-ChRmine-oScarlet 8.7 x10^12^ gc/ml and AAV9- cag- FLOXed-stGtACR2-FusionRed 7.6 x10^12^ gc/ml. The following coordinates were used (anteroposterior (AP), medio-lateral (ML) relative from bregma; dorsoventral from brain surface):

NAC:+1.2AP,+∕−1.3ML,-4.1DV

Nacl:1.55AP,+∕-1.4ML,-4.25DV

VTA:-3.135AP,+∕−0.5ML,-4.4DV

TOS:-1.4AP,+∕-3.28ML,-2.45DV

SNPL:-3.1AP,+∕-2.1ML,-3.6DV

All coordinates are in millimeters. For fluorescence lifetime photometry or optogenetic experiments, an optical fiber (MFC_200/230-0.37_4.5mm_MF1.25_FLT or MFC_200/230-0.37_3mm_MF1.25 mono fiber optic cannula, Doric Lenses) was implanted 100-200 μm above the injection site.

#### Behavior

For all FLIPR measurements, mice were connected to a patch cord that attached to the FLIPR system as in Figures 1 and S1. For fluorescence lifetime baseline recordings, mice were placed in an 8x8 inch black acrylic box. For foot shock experiments, mice were placed in a white plexiglass box with a metal barred floor (ENV-005A, Med Associates ) via which foot shocks can be delivered. Mice received 10-foot shocks (0.5 mA, 500 ms) per session. The foot shock delivery was controlled through a programmable microcontroller (Arduino Uno, Arduino) running a custom script. For food reward experiments, mice were food restricted such that they remained at 80-95% of their initial weight. Mice were given 2-3 g of regular chow daily in addition to the variable number of 20 mg dustless precision chocolate flavor pellets (F05301, Bio Serv). Animals were habituated to an 8x8 inch black acrylic box for 5 – 10 min and received a pellet every minute from an automatic pellet dispenser (ENV-203-20, Med Associates) for a total of 10 pellets. Animal movements were captured by a camera (FL3-U3-13E4M, PointGrey). Bonsai 2.5.1 software controlled the pellet dispenser and was synchronized with the FLIPR MATLAB 2021 software.^57^

#### Histology

Mice were anesthetized with isofluorane inhalation. Mice were perfused transcranially with PBS followed by 4% PFA in PBS. Brains were left overnight in 4% PFA in PBS and then transferred to PBS. Brains were sliced (50-70 μm thickness) using a vibrating blade microtome (Leica Biosystems VT1000S). Slices were blocked using a 5% normal goat serum, 0.1% TritonX-100 PBS solution for two hours at room temperature. Slices were then placed into primary antibody solution (1:1000, 13970, Abcam) overnight. The next day, slices were washed using PBS with 0.1% TritonX-100 (PBST) and placed in a solution with secondary antibody (1:500, A-11039, ThermoFisher) in blocking buffer. Slices were washed with PBST mounted on glass slides using ProLong Diamond Antifade Mountant with DAPI (P36962, ThermoFisher Scientific), and imaged using an Olympus VS200 slide scanning microscope.

#### Pharmacology

D1R antagonist SCH23390 (Tocris, #0925) was diluted in sterile saline and intraperitoneally (IP) injected at 1 mg/ml at 10 mg/kg using a fine syringe (BD 324911, BD Bioscience). Typically, injections were completed 5-20 s after the start of scruffing.

#### Fluorescence lifetime photometry

FLIP was performed using the apparatus shown in Figure S1. A 473 nm pulsed laser (BDS-SM-473-FBC, Becker & Hickl) produced a 50 MHz pulsed laser beam that passed through a Thorlabs polarizer (WPH05ME-488, Thorlabs), and was modulated using an AOM (MTS110-A3-VIS, AA optoelectronic) for power control. AOM attenuation was adjusted by an analog output module (NI-9261, National Instruments) controlled by MATLAB software. The laser beam was centered using a pair of visible coated mirrors (BB1-E01 and KCB1, Thorlabs) and passed through a rotary neutral density filter (NDC-50C-4M-A, Thorlabs) for manual power modulation. The beam reflected on a 488 nm dichroic (Di02-R488-25x36, Semrock, mounted in DFM1-P01, Thorlabs) and was launched into a patch-cord (MFP_200/220/900-0.37_2m_FCM-MF1.25(F)_LAF, Doric) by an additional mirror and an achromatic doublet lens (AC254-050-A-ML, Thorlabs). Emission light was collected using the same fiber implant and patch cord. Emission light was directed through the same lens, mirror (M3) and dichroic. Emission light was filtered through an interference filter (FF01-525/35-25, Idex HS). Thorlabs kinetic magnetic cube (DFM1-P01, Thorlabs) containing a mirror (MGP01-350-700-25x36, Semrock) was used to direct the emission light into a hybrid PMT (HPM-100-07-Cooled, BH) controlled by DCC-100-PCI (BH) for FLIP. An empty Thorlabs kinetic magnetic cube was used to direct the emission light into a GaAsp PMT with improved quantum efficiency (PMT2101, Thorlabs) for FLIPR. For two color FLIPR a red channel was added to the green FLIP and FLIPR set-up, as shown in Figure S6A. Excitation light from a 561nm pulsed laser (BDS-SMY-561, Becker & Hickl) was power modulated using a rotary neutral density filter (NDC-50C-4M-A, Thorlabs) and directed into the patch cord using mirrors (BB1-E01, Thorlabs). Emission light was separated from the green emission fluorescence using a 552nm dichroic beamsplitter (FF552-Di02-25x36, IDEX) and separated from the 561 nm excitation light using a 573 nm dichroic beamsplitter (FF573-Di01-25x36, IDEX). Red emission light was filtered using a 630/60 nm bandpass filter (HHQ630/60, Nikon) and captured using a GaAsp PMT (PMT2101, Thorlabs). All components were light shielded using a variety of Thorlabs lens tubes.

For time domain FLIP, the pulsed laser synchronization port and a hybrid PMT were connected to a time correlated single photon counting system (SPC-830, Becker & Hickl), which detected the time difference between the laser pulse and photon arrival. Data was collected at 1-5 s intervals, with 0.15 s of data transfer time using custom software written in MATLAB 2012a. FLIP data was collected using a rate of 200 kHz–1 MHz accepted photons, except for the data shown in Figure 2E which are shown as a function of variable photon count rates detected by the PMT. Typical excitation power used was 0.5-18 μW. For accuracy comparison experiments, fluorescence lifetime of fluorescent compounds was calculated through single exponential fitting of the photon histogram for each measurement point as previously described.^2,59^ For all other experiments, the average fluorescence lifetime was calculated from the single photon histogram using Equation 1:

(Equation 1) $$\tau =t-t_{0}=\frac{\int tF(t)dt}{\int F(t)dt}-t_{0}$$

wherein τ is the average fluorescence lifetime, t is the average arrival time with respect to the laser pulse, F(t) is the number of photons at time bin t and $t_{0}$ is the timebin corresponding to the peak of the exponentially fitted histogram. The single photon histogram was truncated at 8 – 11.7ns after $t_{0}$ to reject delayed reflection autofluorescence from the system.

For frequency domain FLIPR, the PMT (PMT2101, Thorlabs) and laser synchronization port was connected to custom made analog phase processing unit (Figures 1 and S1A). The laser synchronization pulse was connected to a low-pass filter (BLP-70+, Mini-circuits), 20dB attenuator (HAT-20+, Mini-circuits), low noise amplifier (ZX60-P103LN+, Mini-circuits) and 4-way power splitter (ZSC-4-3+, Mini-circuits). From the power splitter each channel was connected to a phase shifter (TB-JSPHS-51+, Mini Circuits), a channel specific delay line (141- ## BM+, Mini-circuits), a low noise amplifier (ZX60-P103LN+, Mini-circuits), low-pass filter (BLP-70+, Mini-circuits) and LO port of a level 17 frequency mixer (ZX05-1HW-S+, Mini-circuits). The PMT (PMT2101 with internal transimpedance amplifier, Thorlabs) voltage output was connected to a low-pass filter (BLP-70+, Mini-circuits), and a bias tee (ZFBT-4R2G+, Minicircuits), which split the low frequency intensity information (VDC) from the high frequency phase signal (VRF). The VDC was captured using an analog data acquisition module (NI 9215, National Instruments). The VRF signal passed through a low noise amplifier (ZX60-P103LN+, Mini-circuits), and 20dB attenuator (HAT-20+, Mini-circuits). The VRF was connected to a 4-way splitter (ZSC-4-3+, Minicircuits) and connected to the RF port of frequency mixers. The intermediate frequency port of the frequency mixers was connected to bias tee’s (ZFBT-4R2G+, Mini-circuits). The high frequency port of the bias tees was connected to a 30 dB attenuator (HAT-30+, Mini-circuits) and terminated at 50 Ohm using a resistor. The low frequency port of the bias tee was connected to a 1 kHz low-pass filter (EF110, Thorlabs) and a 24-bit analog input module (NI 9239, National instruments). See Table S1 for a detailed list of the components required to build a FLIPR system. Data was collected and processed in real time using custom MATLAB software. Fluorescence lifetime was calculated using Equation 2

(Equation 2) $$\tau =\frac{1}{\omega }\frac{V_{IF}(0)-V_{IF}(\pi )-d13}{V_{IF}(0.5\pi )-V_{IF}(1.5\pi )-d24}$$

wherein τ is the average fluorescence lifetime, ω is the angular frequency, VIF(0−1.5π) are the mixer outputs from phase shifted channel 1-4, d13 and d24 are channel specific offset factors determined during calibration. Although VIF(0π) and VIF(0.5π) are theoretically sufficient to determine the fluorescence lifetime, VIF power dependent offsets in response to increases in photon rates can arise, and compensation by subtraction of VIF(1) and VIF(1.5) allows for more accurate lifetime estimation across photon rates, respectively. Data was averaged using a moving mean and down sampled to the required frequencies.

Fluorescence intensity was calculated using Equation 3:

(Equation 3) $$F=VDC-O_{B}$$

wherein F is intensity signal corrected for baseline voltage, VDC is the voltage coming from the bias tee and $O_{B}$ is the offset of the bias tee determined during calibration.

G and S phasor components were calculated using Equations 4 and 5, respectively.

(Equation 4) $$g=\frac{V_{IF}(0.5\pi )-V_{IF}(1.5\pi )-d_{13}}{2(VDC-O_{B})\left(\frac{M}{B}\right)}$$

(Equation 5) $$s=\frac{V_{IF}(0\pi )-V_{IF}(\pi )-d_{24}}{2(VDC-O_{B})\left(\frac{M}{B}\right)}$$

wherein the M is the mixer conversion loss and B is the bias tee conversion loss, measured through calibration. Lifetimes with a single exponential decay have phasor components located on a semi-circle termed the universal circle (centered at g=0.5, s=0, radius=0.5), while lifetimes with a multi-exponential decay fall outside of the universal circle. Phasor location can be used to decompose complex mixtures of lifetime agents into individual species, such as the ratio between bound and unbound GEFI or GEFI fluorescence and autofluorescence.

Autofluorescence correction was performed using an intensity weighted average function to extract sensor fluorescence lifetime from measured autofluorescence.


(Equation 6) $$\tau_{Observed}=\frac{\tau_{F1}\times I_{F1}+\tau_{F2}\times I_{F2}}{I_{Observed}}$$

Wherein, $\tau_{\mathrm{Observed}}$, $\tau_{F1}$ and $\tau_{F1}$ represent the average, fluorescent source 1 and fluorescent source 2 lifetime respectively, and $I_{\mathrm{Observed}}$, $I_{F1}$ and $I_{F2}$ represent the measured, fluorescent source 1 and fluorescent source 2 intensity, respectively. Autofluorescence correction was performed based on empirically determined levels of autofluorescence caused by fiber implantation, tissue damage and viral transfection in the TOS and NAC (S5). Autofluorescence correction was only performed when comparing across brain regions, as autofluorescence contribution errors in delta lifetime calculations were minimal. To limit autofluorescence, only low autofluorescence fiber implants and patch-cords are recommended when using FLIPR, such as the fiber implants (MFC_200/230-0.37_4.5mm_MF1.25_FLT) and patchcords (MFP_200/220/900-0.37_2m_FCM-MF1.25(F)_LAF, Doric) used in this study.

#### FLIPR calibration

Calibration of the FLIPR system was achieved through the measurement of fluorescence lifetime standard Coumarin 6 (2.5x10^−5^ mg/ml, Sigma Aldrich) dissolved in ethanol which has a known fluorescence lifetime of 2.39-2.41ns. Coumarin 6 solution was measured by FLIPR through a fiber implant of the same length as implanted in animals. As many electronic components and data acquisition systems are temperature sensitive, care should be taken to perform calibration at the temperature that will be used for the experiment. In addition, temperature variation during recordings should be minimized to ensure continued validity of the initial calibration.

The first step in calibrating the system is to determine the control voltages set on the phase shifters ($V_{b}$), so that the phase shifters achieve 0, 0.5, 1 and 1.5 π shift with respect to instantaneous lifetime (0ns). This is achieved by applying a 0-10V ramp over the phase shifters, which allows for the capture of a sinusoidal curve in the VIF(1-4) channels, containing minimal and maximal values. The phase calibration curve can be extracted per channel from the VIF data, by

(Equation 7) $$\varphi (V_{b})=\arcsin\left(\frac{2\times VIF(V_{b})-(VIF_{max}+VIF_{min})}{VIF_{max}-VIF_{min}}\right)-\arctan\;(\tau \omega )$$

Wherein, τ equals the lifetime of the coumarin 6, and ω the angular frequency of the laser. The phase calibration curve is fitted using a polynomial fit, to reduce the impact of electrical noise. From the phase calibration curve the bias voltage ($V_{b}$) over individual phase shifters can be determined.

Next, voltage offsets over the VIF and VDC channels are determined. The shutter in front of the PMT is closed and data is collected across all channels. Here the voltage offset over VDC ($O_{B}$) and VIF ($O_{m}$) channels are

(Equation 8) $$O_{B}=\bar{VDC}$$

(Equation 9) $$O_{m,i}=VIF_{i}=O(P_{RF})+d_{i}$$

Wherein $O(P_{\mathrm{RF}})$ and $d_{i}$ represent the intensity dependent and independent offset. $O(P_{\mathrm{RF}})$ is the same across channels. Therefore, calibration factors $d_{13}$ and $d_{24}$ can be extracted, by

(Equation 10) $$d_{13}=d_{1}-d_{3}=VIF_{1}-VIF_{3}$$

(Equation 11) $$d_{24}=d_{2}-d_{4}=VIF_{2}-VIF_{4}$$

The control voltages set on the phase shifters ($V_{b}$) are then re-determined taking into account the known offsets, as described above. Finally, the ratio of conversion loss in the mixer and bias tee (M/B) is determined to allow for phasor calculation.


(Equation 12) $$\frac{M}{B}=\frac{VIF_{max}-VIF_{min}}{2(VDC-O_{b})}\sqrt{1+{\left(\tau \omega \right)}^{2}}$$

See Zhang et al. for more details about the underlying theoretical principles of FLIPR calibration.^19^

#### Conversion from lifetimes to state occupancy and ligand concentration

Use of absolute lifetime measurements (i.e. in units of time) to obtain a quantitative understanding of the sensor state is possible. We consider the case of a sensor that exists in two states, $s_{1}$ and $s_{2}$, which could correspond to the dopamine free and dopamine bound states of dLight3.8 or the unphosphorylated and phosphorylated states of FLIM-AKAR. Define:

$\tau_{1}$ and $\tau_{2}$ as the lifetimes of $s_{1}$ and $s_{2}$

f as the fraction of sensors in state $s_{1}$ (with 1−f the fraction in state $s_{2}$)

Define fluorescence intensity as a function of the fraction of sensors in $s_{1}$ normalized to the fluorescence when all the sensors are in $s_{1}$ (i.e., f=1) (Equation 13):

(Equation 13) F(f)=f+R(1−f)=f(1−R)+R

which naturally ranges from 1 to R as f ranges from 1 to 0. Here R is the ratio of sensor fluorescence intensity when all of the sensors are in state $s_{2}$ compared to $s_{1}$:

(Equation 14) $$R=\frac{F(0)}{F(1)}$$

Assuming that $s_{2}$ is brighter than $s_{1}$ (i.e., R>1), F corresponds to a delta-F/F0 measurement.

To convert F to fluorescence intensity measured by the device ($F^{\prime }$), an additional scale factor (s) is necessary that converts to units measured by the DAQ (typically volts) and whose value depends on illumination intensity, concentration of sensor, detector efficiency, and more. This produces (Equation 15):

(Equation 15) $$F^{\prime }(f)=sF(f)=s(f(1-R)+R)$$

The average fluorescence lifetime of a population of sensors is (Equation 16):

(Equation 16) $$\tau (f)=\frac{f\tau_{1}+R(1-f)\tau_{2}}{f+(1-f)f_{2}}=\frac{f(\tau_{1}-R\tau_{2})+R\tau_{2}}{f(1-R)+R}=\frac{f(\tau_{1}-R\tau_{2})+R\tau_{2}}{F(f)}$$

which is simply the mean of the lifetimes of each state weighted by the number or fraction of photons emitted by sensors in each state.

Using FLIPR corresponds to collect many samples of $F^{\prime }$ and τ simultaneously, which are each a function on a common underlying and unknown variable f. If n time samples are collected, then we can use the 2n data points ($F_{1}^{\prime },\ldots ,F_{n}^{\prime }$ and $\tau_{1},\ldots ,\tau_{n}$) to fit Equations 15 and 16 simultaneously which contain n + 4 free variables ($f_{1},\ldots ,f_{n}$ plus $\tau_{1}$, $\tau_{2}$, R, and s). In practice, s is time dependent because bleaching affects its value. Therefore, the fit should be done with small snippets of data during which bleaching is minimal. We used 40 sec stretches (4000 data points sampled at 100 Hz) and performed fits with the lsqnonlin optimizer in Matlab. See github for sample code which was used to produce the fit in Figure 4B. In practice, it is convenient to fix known values of the 4 parameters based on independent measurements. For example, based on dopamine depletion and receptor antagonism experiments, we know that *in vivo*, $\tau_{1}$, corresponding to the lifetime of dopamine free dLight3.8, is 1.55 ns.

As a last step, although one that is potentially too unconstrained to be useful, for a sensor of a ligand L (i.e., dopamine for dLight3.8) f can be converted to [L] using:

(Equation 17) $$[L]=K_{D}\frac{1-f}{f}$$

dLight3.8 has a $K_{D}$ of 148nM *in vitro*^34^. However, in practice, the value of$K_{D}$ *in vivo* is difficult to obtain.

The above fitting procedure is relatively under constrained unless data from a wide dynamic range of the sensor states (i.e., f ranging from much of 0 to 1) is used such that the non-linearities of Equation 15 and 16 become manifested. Therefore, we prefer to continue to express FLIPR measurements in units of time, which, if a well calibrated system is used, can be compared directly across time, preparations, and laboratories.

#### Optogenetic manipulation

To excite ChRmine, a 635 nm laser (Optoengine) was modulated using an AOM at 15 Hz, for 500 ms with 5 ms pulsewidth at 1.1, 1.9 and 2.4 mW. To excite stGtACR2, a blue 488 nm laser (GEM 473, Laser Quantum) was modulated using an AOM for a duration of 5 seconds continuous illumination at 6mW. Light was delivered to the targeted brain region using a 200 μm Doric patch cord. Laser light power was calibrated at the tip of the patch cord using a Thorlabs digital optical power meter (PM400 and S120C).

#### ACSF and brain slice preparation

Artificial cerebrospinal fluid (ACSF) was prepared as followed: 125 mM NaCl, 2.5 mM KCl, 25 mM NaHCO3, 2 mM CaCl2, 1 mM MgCl2, 1.25 mM NaH2PO4 and 11 mM glucose (300mOsm/kg). Choline-based solution was prepared as followed: 110 mM choline chloride, 25 mM NaHCO3, 2.5 mM KCl, 7 mM MgCl2, 0.5 mM CaCl2, 1.25 mM NaH2PO4, 25 mM glucose, 11.6 mM sodium ascorbate, and 3.1 mM sodium pyruvate (320 mOsm/kg).

The mouse was perfused using ice cold ACSF and the brain was immediately extracted and placed in Leica vibratome slicing chamber in ice cold ACSF. Brain slices of 300 μm were taken and incubated in a chamber containing choline-based solution at 34-degree Celsius. After 10 minutes the brain slices were transferred to a chamber with ACSF at 34-degree Celsius. After 1 hour the slices were left at room temperature, before being transferred for fluorescence lifetime microscopy (FLIM). During FLIM, the slices were held at 32-34 degrees Celsius in ACSF. All solutions were continuously bubbled with carbogen.

#### Fluorescence lifetime microscopy (FLIM)

Lifetime measurement of brain slices were performed using a custom-built 2-photon microscope. dLight3.8mut fluorophores in brain slices were imaged at 920nm excitation light using a Ti:Sapphire laser (Chameleon Vision II, 80 MHz, Coherent, Santa Clara, CA). Emission light was collected using a photon multiplier tube (H7422-40MOD, Hamamatsu). Counting data of individual photons were collected using a vDAQ system (MBF Bioscience, Ashburn, VA) through ScanImage v2021.0.0 in MATLAB 2021b. Photons were binned at 0.391ns for 31 bins per pulse, relative to the reference laser pulse. Time bin 32 was utilized to count the total number of photons per pixel, to allow for real-time intensity imaging.

Data was collected at 1x zoom, 1.07 Hz, at 512 pixels per line for 60 frames. The lifetime of each frame was calculated using Equation 18.


(Equation 18) $$\tau =\frac{\Sigma F(t)*t}{\Sigma F(t)}$$

Where F(t) is the number of photons in each 0.391 ns time bin, and t is the corresponding time bin. Sites of 128 by 128 pixels were selected based on sensor expression, and lifetime was determined by averaging the fluorescence lifetime across frames. D1R antagonist SCH23390 (Final concentration 10μM, Tocris, #0925) in ACSF was added to the solution after baseline recording. Brain slices were incubated with D1R antagonist for 10 minutes before imaging.

### QUANTIFICATION AND STATISTICAL ANALYSIS

Data was analyzed using custom scripts in MATLAB (v2021a and v2024a) and Python (v3.8) and using the data analysis toolbox in excel (v2410). No statistical sample size pre-calculation was performed. Experimenters were not blinded to animal groups, as pilot sensor characterization clearly distinguished dLight3.8 from dLight3.8mut and fiber placement TOS and NAC. Paired sample T-test, two sample T-test and one-way repeated measures ANOVA statistical tests were applied to data as indicated in figure legends. No multiple comparison correction was performed as none of the multiple comparison tests were significant. Signal to noise ratio was calculated by dividing the mean peak signal by the mean baseline standard deviation. In figures, single star (*), double star (**) and triple star (***), represent p < 0.05, p<0.01 and p<0.001, respectively.

## Supplementary Material

MMC2

MMC1

Supplemental information can be found online at https://doi.org/10.1016/j.neuron.2025.08.013.

## Figures and Tables

**Table:** KEY RESOURCES TABLE

REAGENT or RESOURCE	SOURCE	IDENTIFIER
Antibodies		
Chicken polyclonal anti-GFP	Abcam	Cat.#: 13970; RRID:AB_300798
Goat anti-Chicken IgY (H+L), Alexa Fluor^™^ 488	ThermoFisher	Cat.#: A-11039; RRID: AB_2534096
Bacterial and virus strains		
AAV9-CAG-dLight3.8	UNC Neurotools	AAV in stock
AAV9-hSyn-dLight3.8mut	This paper	Available upon request
AAV9-hsyn-sfGFP	This paper	Available upon request
AAV9-hsyn-BrUSLEE	This paper	Available upon request
AAV9-hsyn-Ivy	This paper	Available upon request
AAV2-FLEX-FLIM-AKART391A	Chen et al.^2^	Addgene #60446
AAV1-syn-FLEX.NES-jRCaMP1b	Dana et al.^38^	Addgene #100850
pAAV-nEF-Con/Foff 2.0-ChRmine-oScarlet	Fenno et al.^35^	Addgene #137161
AAV9-cag-FLOXed-stGtACR2-FusionRed rev. WPRE	Silvia Arber Lab	N/A
Chemicals, peptides, and recombinant proteins		
Fluorescein	Sigma-Aldrich	Cat.#: F2456-100G
Coumarin 6	Sigma-Aldrich	Cat.#: 442631-1G
Acridine Orange	Invitrogen	Cat.#: 2488800
Potassium Iodide	Honeywell	Cat.#: 7681-11-0
D1R antagonist SCH23390	Tocris	Cat.#: 0925
Deposited data		
FLIP and FLIPR lifetime photometry data	This paper	Available at zenodo data: https://zenodo.org/records/16753803; https://doi.org/10.5281/zenodo.16753802
Experimental models: Organisms/strains		
Mouse: C57BL/6J	Jackson Laboratory	RRID: IMSR_JAX:000664
Mouse: DAT-cre	Jackson Laboratory	RRID:IMSR_JAX:006660
Mouse: Drd1a-cre	MMRRC UC Davis	RRID:MMRRC_030989-UCD; B6.FVB(Cg)-Tg(Drd1-cre)EY262Gsat/Mmucd
Software and algorithms		
MATLAB	Mathworks	https://www.mathworks.com/; RRID:SCR_001622
ScanImage (2012a version)	Bernardo Sabatini	https://github.com/bernardosabatini/SabalabAcq
ScanImage (2021a version)	Bernardo Sabatini	https://github.com/bernardosabatini/SabalabAcq
FLIPR acquisition software	This paper	https://github.com/Bartlodder/FLIPRMatlabSoftware
FLIPR analysis code	This paper	https://github.com/Bartlodder/FLIPR_data_analysis; https://doi.org/10.5281/zenodo.16753893
Bonsai-rx	Lopes et al.^57^	https://bonsai-rx.org/; RRID:SCR_021512
Other		
Chocolate Pellets	BioServ	Cat.#: F05301
Pellet Dispenser	MedAssociates	Cat.#: ENV-203-20
552nm dichroic beamsplitter	IDEX	Cat.#: FF552-Di02-25x36
573 nm dichroic beamsplitter	IDEX	Cat.#: FF573-Di01-25x36
630/60 nm bandpass filter	Nikon	Cat.#: HHQ630/60
GaAsp Photomultiplier Tube	Thorlabs	Cat.#: PMT2101
Rotary neutral density filter	Thorlabs	Cat.#: NDC-50C-4M-A
Mirrors	Thorlabs	Cat.#: BB1-E01
Optical power meter console	Thorlabs	Cat.#: PM400
Photodiode power sensor	Thorlabs	Cat.#: S120C
FLIM data-acquisition board	Mbf bioscience	Cat.#: vDAQ-HS
Fiber implants	Doric Lenses	Cat.# MFC_200/230-0.37_4.5mm_MF1.25_FLT
Fiber optic patch cord	Doric Lenses	MFP_200/220/900-0.37_3m_FCM-MF1.25
Footshock apparatus	MedAssociates	Cat.#: ENV-005A
Camera	PointGrey	Cat.#: FL3-U3-13E4M
635 nm laser	OptoEngine	Cat.#: MRL-III-635L
Acousto-optic modulator	AA optoelectronic	Cat.#: MTS110-A3-VIS
470 nm laser	Laser Quantum	Cat.#: GEM 473
Arduino Uno Rev3	Arduino	Cat.#: A000066

## Highlights

- FLIPR enables high-speed (kHz) fluorescence lifetime measurements with ps precision
- FLIPR measures neuronal signals in absolute units to compare across time and mice
- FLIPR enables measurement of absolute phasic and tonic neuromodulator levels
- Tonic and phasic dopamine across striatal subregions are differentially modulated

## References

[1] Yang Y, Li B, and Li Y (2024). Genetically Encoded Sensors for the In Vivo Detection of Neurochemical Dynamics. Annu. Rev. Anal. Chem. (Palo Alto. Calif) 17, 367–392. 10.1146/annurev-anchem-061522-044819. 38639991

[2] Chen Y, Saulnier JL, Yellen G, and Sabatini BL (2014). A PKA activity sensor for quantitative analysis of endogenous GPCR signaling via 2-photon FRET-FLIM imaging. Front. Pharmacol 5, 56. 10.3389/fphar.2014.00056. 24765076 PMC3980114

[3] Jing M, Li Y, Zeng J, Huang P, Skirzewski M, Kljakic O, Peng W, Qian T, Tan K, Zou J, (2020). An optimized acetylcholine sensor for monitoring in vivo cholinergic activity. Nat. Methods 17, 1139–1146. 10.1038/s41592-020-0953-2. 32989318 PMC7606762

[4] Patriarchi T, Cho JR, Merten K, Howe MW, Marley A, Xiong WH, Folk RW, Broussard GJ, Liang R, Jang MJ, (2018). Ultrafast neuronal imaging of dopamine dynamics with designed genetically encoded sensors. Science 360, eaat4422. 10.1126/science.aat4422. 29853555 PMC6287765

[5] Zhang Y, Rózsa M, Liang Y, Bushey D, Wei Z, Zheng J, Reep D, Broussard GJ, Tsang A, Tsegaye G, (2023). Fast and sensitive GCaMP calcium indicators for imaging neural populations. Nature 615, 884–891. 10.1038/s41586-023-05828-9. 36922596 PMC10060165

[6] Tian L, Hires SA, Mao T, Huber D, Chiappe ME, Chalasani SH, Petreanu L, Akerboom J, McKinney SA, Schreiter ER, (2009). Imaging neural activity in worms, flies and mice with improved GCaMP calcium indicators. Nat. Methods 6, 875–881. 10.1038/NMETH.1398. 19898485 PMC2858873

[7] Dong C, Gowrishankar R, Jin Y, He XJ, Gupta A, Wang H, Sayar-Atasoy N, Flores RJ, Mahe K, Tjahjono N, (2024). Unlocking opioid neuropeptide dynamics with genetically encoded biosensors. Nat. Neurosci 27, 1844–1857. 10.1038/s41593-024-01697-1. 39009835 PMC11374718

[8] Cui G, Jun SB, Jin X, Pham MD, Vogel SS, Lovinger DM, and Costa RM (2013). Concurrent activation of striatal direct and indirect pathways during action initiation. Nature 494, 238–242. 10.1038/nature11846. 23354054 PMC4039389

[9] Lee SJ, Chen Y, Lodder B, and Sabatini BL (2019). Monitoring behaviorally induced biochemical changes using fluorescence lifetime photometry. Front. Neurosci 13, 766. 10.3389/fnins.2019.00766. 31417343 PMC6685078

[10] Simpson EH, Akam T, Patriarchi T, Blanco-Pozo M, Burgeno LM, Mohebi A, Cragg SJ, and Walton ME (2024). Lights, fiber, action! A primer on in vivo fiber photometry. Neuron 112, 718–739. 10.1016/j.neuron.2023.11.016. 38103545 PMC10939905

[11] Yellen G, and Mongeon R (2015). Quantitative two-photon imaging of fluorescent biosensors. Curr. Opin. Chem. Biol 27, 24–30. 10.1016/J.CBPA.2015.05.024. 26079046 PMC4553104

[12] Koveal D, Díaz-García CM, and Yellen G (2020). Fluorescent Biosensors for Neuronal Metabolism and the Challenges of Quantitation. Curr. Opin. Neurobiol 63, 111–121. 10.1016/J.CONB.2020.02.011. 32559637 PMC7646541

[13] Sherathiya VN, Schaid MD, Seiler JL, Lopez GC, and Lerner TN (2021). GuPPy, a Python toolbox for the analysis of fiber photometry data. Sci. Rep 11, 24212. 10.1038/S41598-021-03626-9. 34930955 PMC8688475

[14] Bianco M, Balena A, Pisanello M, Pisano F, Sileo L, Spagnolo B, Montinaro C, Sabatini BL, Vittorio MD, and Pisanello F (2021). Comparative study of autofluorescence in flat and tapered optical fibers towards application in depth-resolved fluorescence lifetime photometry in brain tissue. Biomed. Opt. Express 12, 993–1010. 10.1364/BOE.410244. 33680555 PMC7901336

[15] Zhang W-T, Chao T-HH, Yang Y, Wang T-W, Lee S-H, Oyarzabal EA, Zhao J, Nonneman R, Pegard NC, Zhu H, (2022). Spectral fiber photometry derives hemoglobin concentration changes for accurate measurement of fluorescent sensor activity. Cell Rep. Methods 2, 100243. 10.1016/j.crmeth.2022.100243. 35880016 PMC9308135

[16] Liang Z, Ma Y, Watson GDR, and Zhang N (2017). Simultaneous GCaMP6-based fiber photometry and fMRI in rats. J. Neurosci. Methods 289, 31–38. 10.1016/J.JNEUMETH.2017.07.002. 28687521 PMC5582003

[17] Ma P, Chen P, Tilden E, Aggarwal S, Oldenborg A, and Chen Y (2024). Fast and slow: Recording neuromodulator dynamics across both transient and chronic time scales. Sci. Adv 10, eadi0643. 10.1126/sciadv.adi0643. 38381826 PMC10881037

[18] Ma P, Sternson S, and Chen Y (2024). The promise and peril of comparing fluorescence lifetime in biology revealed by simulations. eLife 13, RP101559. 10.7554/eLife.101559 PMC12367299 40832998

[19] Zhang Y, Guldner IH, Nichols EL, Benirschke D, Smith CJ, Zhang S, and Howard SS (2021). Instant FLIM enables 4D in vivo lifetime imaging of intact and injured zebrafish and mouse brains. Optica 8, 885–897. 10.1364/OPTICA.426870. 39867356 PMC11759494

[20] Lodder B, Lee SJ, and Sabatini BL (2021). Real-Time, In Vivo Measurement of Protein Kinase A Activity in Deep Brain Structures Using Fluorescence Lifetime Photometry (FLiP). Curr. Protoc 1, e265. 10.1002/CPZ1.265. 34661994 PMC8650723

[21] Vu CQ, and Arai S (2023). Quantitative Imaging of Genetically Encoded Fluorescence Lifetime Biosensors. Biosensors 13, 939. 10.3390/bios13100939. 37887132 PMC10605767

[22] Harvey CD, Ehrhardt AG, Cellurale C, Zhong H, Yasuda R, Davis RJ, and Svoboda K (2008). A genetically encoded fluorescent sensor of ERK activity. Proc. Natl. Acad. Sci. USA 105, 19264–19269. 10.1073/pnas.0804598105. 19033456 PMC2614750

[23] Ma L, Jongbloets BC, Xiong WH, Melander JB, Qin M, Lameyer TJ, Harrison MF, Zemelman BV, Mao T, and Zhong H (2018). A Highly Sensitive A-Kinase Activity Reporter for Imaging Neuromodulatory Events in Awake Mice. Neuron 99, 665–679.e5. 10.1016/j.neuron.2018.07.020. 30100256 PMC6152931

[24] Farrants H, Shuai Y, Lemon WC, Monroy Hernandez C, Zhang D, Yang S, Patel R, Qiao G, Frei MS, Plutkis SE, (2024). A modular chemigenetic calcium indicator for multiplexed in vivo functional imaging. Nat. Methods 21, 1916–1925. 10.1038/s41592-024-02411-6. 39304767 PMC11466818

[25] van der Linden FH, Mahlandt EK, Arts JJG, Beumer J, Puschhof J, de Man SMA, Chertkova AO, Ponsioen B, Clevers H, van Buul JD, (2021). A turquoise fluorescence lifetime-based biosensor for quantitative imaging of intracellular calcium. Nat. Commun 12, 7159. 10.1038/S41467-021-27249-W. 34887382 PMC8660884

[26] Cui G, Jun SB, Jin X, Luo G, Pham MD, Lovinger DM, Vogel SS, and Costa RM (2014). Deep brain optical measurements of cell typespecific neural activity in behaving mice. Nat. Protoc 9, 1213–1228. 10.1038/nprot.2014.080. 24784819 PMC4100551

[27] Malacrida L, Ranjit S, Jameson DM, and Gratton E (2021). The Phasor Plot: A Universal Circle to Advance Fluorescence Lifetime Analysis and Interpretation. Annu. Rev. Biophys 50, 575–593. 10.1146/annurev-biophys-062920-063631. 33957055

[28] Henderson BC (1981). Mixers: Part 1 Characteristics and Performance (Watkins-Johnson Company).

[29] Mini-circuits. (2015). Pairing Mixers with Reflectionless Filters to Improve System Performance. https://www.minicircuits.com/app/AN75-007.pdf?srsltid=AfmBOormozt0s4L30xSMkiB03PeXlbW9z9y87AiRE8NjaKFu1Y9VEqiB.

[30] Lee SJ, Lodder B, Chen Y, Patriarchi T, Tian L, and Sabatini BL (2021). Cell-type-specific asynchronous modulation of PKA by dopamine in learning. Nature 590, 451–456. 10.1038/S41586-020-03050-5. 33361810 PMC7889726

[31] Pédelacq JD, Cabantous S, Tran T, Terwilliger TC, and Waldo GS (2006). Engineering and characterization of a superfolder green fluorescent protein. Nat. Biotechnol 24, 79–88. 10.1038/nbt1172. 16369541

[32] Mamontova AV, Solovyev ID, Savitsky AP, Shakhov AM, Lukyanov KA, and Bogdanov AM (2018). Bright GFP with subnanosecond fluorescence lifetime. Sci. Rep 8, 13224. 10.1038/s41598-018-31687-w. 30185895 PMC6125319

[33] Slubowski CJ, Funk AD, Roesner JM, Paulissen SM, and Huang LS (2015). Plasmids for C-terminal tagging in Saccharomyces cerevisiae that contain improved GFP proteins, Envy and Ivy. Yeast 32, 379–387. 10.1002/YEA.3065. 25612242 PMC4390471

[34] Tian L, Roshgadol J, Chouinard J, Majumder S, Scott E, Borges K, Hagihara K, Mancini N, Stevenson T, Kamath T, (2025). Sensitive dLight for imaging broad-spectrum dopamine events across brain regions. Research Square. 10.21203/rs.3.rs-7313638/v1.

[35] Fenno LE, Ramakrishnan C, Kim YS, Evans KE, Lo M, Vesuna S, Inoue M, Cheung KYM, Yuen E, Pichamoorthy N, (2020). Comprehensive Dual- and Triple-Feature Intersectional Single-Vector Delivery of Diverse Functional Payloads to Cells of Behaving Mammals. Neuron 107, 836–853.e11. 10.1016/J.NEURON.2020.06.003. 32574559 PMC7687746

[36] Menegas W, Akiti K, Amo R, Uchida N, and Watabe-Uchida M (2018). Dopamine neurons projecting to the posterior striatum reinforce avoidance of threatening stimuli. Nat. Neurosci 21, 1421–1430. 10.1038/s41593-018-0222-1. 30177795 PMC6160326

[37] Watabe-Uchida M, Eshel N, and Uchida N (2017). Neural Circuitry of Reward Prediction Error. Annu. Rev. Neurosci 40, 373–394. 10.1146/annurev-neuro-072116-031109. 28441114 PMC6721851

[38] Dana H, Mohar B, Sun Y, Narayan S, Gordus A, Hasseman JP, Tsegaye G, Holt GT, Hu A, Walpita D, (2016). Sensitive red protein calcium indicators for imaging neural activity. eLife 5, e12727. 10.7554/eLife.12727. 27011354 PMC4846379

[39] Becker W. (2023). The bh TCSPC Handbook, Tenth Edition (Becker and Hickl GmbH).

[40] Rodeberg NT, Sandberg SG, Johnson JA, Phillips PEM, and Wightman RM (2017). Hitchhiker’s Guide to Voltammetry: Acute and Chronic Electrodes for In Vivo Fast-Scan Cyclic Voltammetry. ACS Chem. Neurosci 8, 221–234. 10.1021/acschemneuro.6b00393. 28127962 PMC5783156

[41] Ariansen JL, Heien MLAV, Hermans A, Phillips PEM, Hernadi I, Bermudez MA, Schultz W, and Wightman RM (2012). Monitoring extracellular pH, oxygen, and dopamine during reward delivery in the striatum of primates. Front. Behav. Neurosci 6, 36. 10.3389/fnbeh.2012.00036. 22783176 PMC3389715

[42] Nicolai EN, Michelson NJ, Settell ML, Hara SA, Trevathan JK, Asp AJ, Stocking KC, Lujan JL, Kozai TDY, and Ludwig KA (2018). Design choices for next-generation neurotechnology can impact motion artifact in electrophysiological and fast-scan cyclic voltammetry measurements. Micromachines 9, 494. 10.3390/mi9100494. 30424427 PMC6215211

[43] Lucio Boschen S, Trevathan J, Hara SA, Asp A, and Lujan JL (2021). Defining a Path Toward the Use of Fast-Scan Cyclic Voltammetry in Human Studies. Front. Neurosci 15, 728092. 10.3389/fnins.2021.728092. 34867151 PMC8633532

[44] Robbins EM, Castagnola E, and Cui XT (2022). Accurate and stable chronic in vivo voltammetry enabled by a replaceable subcutaneous reference electrode. iScience 25, 104845. 10.1016/j.isci.2022.104845. 35996579 PMC9391596

[45] Gottschalk A, Menees H, Bogner C, Zewde S, Jibin J, Gamam A, Flink D, Mosissa M, Bonneson F, Wehelie H, (2024). Wideband ratiometric measurement of tonic and phasic dopamine release in the striatum. Preprint at bioRxiv. 10.1101/2024.10.17.618918.

[46] Chefer VI, Thompson AC, Zapata A, and Shippenberg TS (2009). Overview of brain microdialysis. Curr. Protoc. Neurosci Chapter 7, Unit7.1. 10.1002/0471142301.ns0701s47. PMC2953244 19340812

[47] Lahiri AK, and Bevan MD (2020). Dopaminergic Transmission Rapidly and Persistently Enhances Excitability of D1 Receptor-Expressing Striatal Projection Neurons. Neuron 106, 277–290.e6. 10.1016/j.neuron.2020.01.028. 32075716 PMC7182485

[48] Speranza L, Di Porzio U, Viggiano D, de Donato A, and Volpicelli F (2021). Dopamine: The neuromodulator of long-term synaptic plasticity, reward and movement control. Cells 10, 735. 10.3390/cells10040735. 33810328 PMC8066851

[49] Dreyer JK, Herrik KF, Berg RW, and Hounsgaard JD (2010). Influence of phasic and tonic dopamine release on receptor activation. J. Neurosci 30, 14273–14283. 10.1523/JNEUROSCI.1894-10.2010. 20962248 PMC6634758

[50] Wise RA, and Robble MA (2020). Dopamine and addiction. Annu. Rev. Psychol 71, 79–106. 10.1146/annurev-psych-010418-103337. 31905114

[51] Grace AA (2016). Dysregulation of the dopamine system in the pathophysiology of schizophrenia and depression. Nat. Rev. Neurosci 17, 524–532. 10.1038/nrn.2016.57. 27256556 PMC5166560

[52] Véronneau-Veilleux F, Robaey P, Ursino M, and Nekka F (2022). A mechanistic model of ADHD as resulting from dopamine phasic/tonic imbalance during reinforcement learning. Front. Comput. Neurosci 16, 849323. 10.3389/fncom.2022.849323. 35923915 PMC9342605

[53] Guthrie M, Myers CE, and Gluck MA (2009). A neurocomputational model of tonic and phasic dopamine in action selection: A comparison with cognitive deficits in Parkinson’s disease. Behav. Brain Res 200, 48–59. 10.1016/j.bbr.2008.12.036. 19162084 PMC4334387

[54] Romero Pinto S, and Uchida N (2023). Tonic dopamine and biases in value learning linked through a biologically inspired reinforcement learning model. Preprint at bioRxiv. 10.1101/2023.11.10.566580. PMC12350682 40804043

[55] de Jong JW, Afjei SA, Pollak Dorocic I, Peck JR, Liu C, Kim CK, Tian L, Deisseroth K, and Lammel S (2019). A Neural Circuit Mechanism for Encoding Aversive Stimuli in the Mesolimbic Dopamine System. Neuron 101, 133–151.e7. 10.1016/j.neuron.2018.11.005. 30503173 PMC6317997

[56] Bäckman CM, Malik N, Zhang YJ, Shan L, Grinberg A, Hoffer BJ, Westphal H, and Tomac AC (2006). Characterization of a mouse strain expressing Cre recombinase from the 3′ untranslated region of the dopamine transporter locus. Genesis 44, 383–390. 10.1002/DVG.20228. 16865686

[57] Lopes G, Bonacchi N, Frazão J, Neto JP, Atallah BV, Soares S, Moreira L, Matias S, Itskov PM, Correia PA, (2015). Bonsai: An event-based framework for processing and controlling data streams. Front. Neuroinform 9, 7. 10.3389/FNINF.2015.00007/BIBTEX. 25904861 PMC4389726

[58] Gerfen CR, Paletzki R, and Heintz N (2013). GENSAT BAC Cre-Recombinase Driver Lines to Study the Functional Organization of Cerebral Cortical and Basal Ganglia Circuits. Neuron 80, 1368–1383. 10.1016/J.NEURON.2013.10.016. 24360541 PMC3872013

[59] Yasuda R, Harvey CD, Zhong H, Sobczyk A, Van Aelst L, and Svoboda K (2006). Supersensitive Ras activation in dendrites and spines revealed by two-photon fluorescence lifetime imaging. Nat. Neurosci 9, 283–291. 10.1038/nn1635. 16429133

