# Supplementary material for: Absolute measurement of fast and slow neuronal signals with fluorescence lifetime photometry at high temporal resolution
Source: Neuron. Author manuscript; Available in PMC 2025 Oct 10. (PMC12509219; doi:10.1016/j.neuron.2025.08.013)
Supplement: MMC1 [file NIHMS2109482-supplement-MMC1.pdf]

Neuron, Volume *113*

Supplemental information

Absolute measurement of fast and slow neuronal signals with fluorescence lifetime photometry at high temporal resolution

Bart Lodder, Tarun Kamath, Ecaterina Savenco, Berend Röring, Michelle Siegel, Julie A. Chouinard, Suk Joon Lee, Caroline Zagoren, Paul Rosen, Isa Hartman, Joshua Timmins, Roger Adan, Lin Tian, and Bernardo L. Sabatini

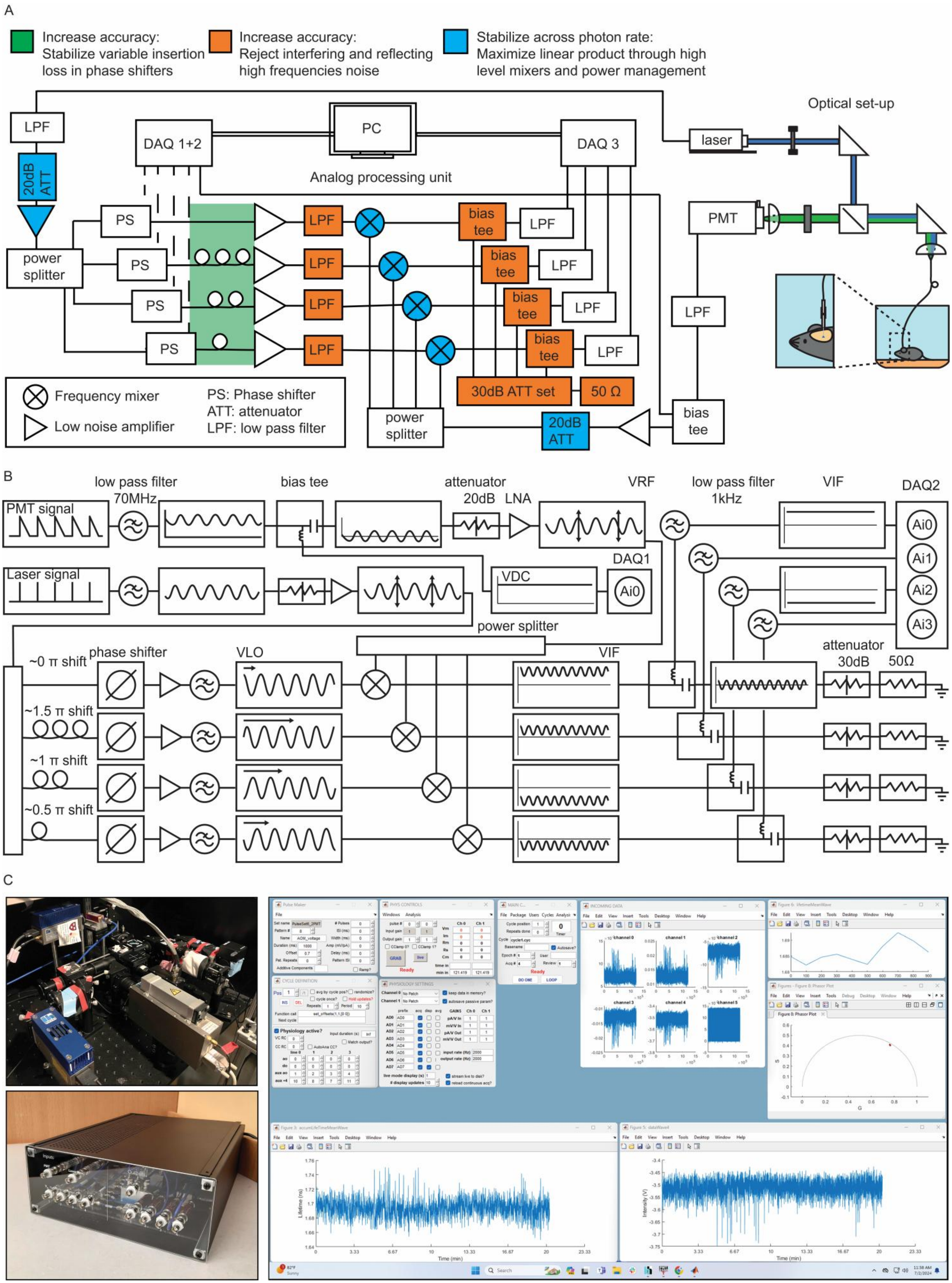
A
Increase accuracy:
Stabilize variable insertion loss in phase shifters
Increase accuracy:
Reject interfering and reflecting high frequencies noise
Stabilize across photon rate:
Maximize linear product through high level mixers and power management
LPF
20dB ATT
DAQ 1+2
PC
DAQ 3
laser
Optical set-up
Analog processing unit
PMT
PS
power splitter
bias tee
30dB ATT set
50 Ω
20dB ATT
Frequency mixer
Low noise amplifier
PS: Phase shifter
ATT: attenuator
LPF: low pass filter
B
PMT signal
low pass filter 70MHz
bias tee
attenuator 20dB
LNA
VRF
low pass filter 1kHz
VIF
DAQ2
Ai0
Ai1
Ai2
Ai3
Laser signal
VDC
DAQ1
power splitter
~0 π shift
phase shifter
VLO
~1.5 π shift
~1 π shift
~0.5 π shift
attenuator 30dB
50Ω
C

Figure S1. FLIPR system detailed diagram, Related to Figure 1

A, FLIPR consists of an optical path (right) and analog processing unit (left). Excitation light is provided by a 50 MHz pulsed 473 nm laser whose output is focused into a fiber optic that couples to a fiber optic stub implanted in the brain. The same fiber optic collects green fluorescence which is separated from the excitation light using a dichroic filter and directed to a PMT. The analog processing unit performs high-speed phase detection using phase shifting and mixing circuitry. Blue, green and orange highlight critical improvements to stabilize and increase the accuracy of fluorescence lifetime calculations across power levels. LPF: low pass filter; ATT: electrical attenuator; LNA: low noise amplifier; PS: phase shifter; DAQ: data acquisition system; TA: transimpedance amplifier; PMT: photon multiplier tube.

B, Schematic of signal processing in the FLIPR computing unit. In the top left, a schematized signal from the PMT and laser reference signal is passed through a low pass filter to extract the 50 MHz modulated signal. The PMT signal is split by a bias tee into a high frequency signal (50 MHz) and DC or intensity (VDC) signal. The high frequency PMT signal (VRF) is power adjusted by an attenuator and low noise amplifier (LNA), and passed into the frequency mixers. The laser reference signal is power adjusted by an attenuator and LNA and split into 4 different channels by a power splitter. The 4 channels are shifted by 0, 0.5, 1 and 1.5 π through physical delay lines and controllable phase shifters, amplified using LNAs and low pass filtered at 70 MHz. The laser reference signal (VLO) channels are passed into the frequency mixers, where multiplication with VRF produces a low frequency (DC-kHz) and high frequency (~100 MHz) VIF signal. High frequency signals are filtered out and absorbed through bias tees, attenuators and 50 Ohm resistors. Low frequency information is low pass filtered at 1 kHz. All relevant signals are collected by a data acquisition system (DAQ). VLO: local oscillator voltage; VRF: radio frequency voltage; VIF: intermediate frequency voltage. VDC: direct current voltage.

C, Image of the optical system (*top left*), housed analog processing unit (*bottom left*) and FLIPR software (*right*).

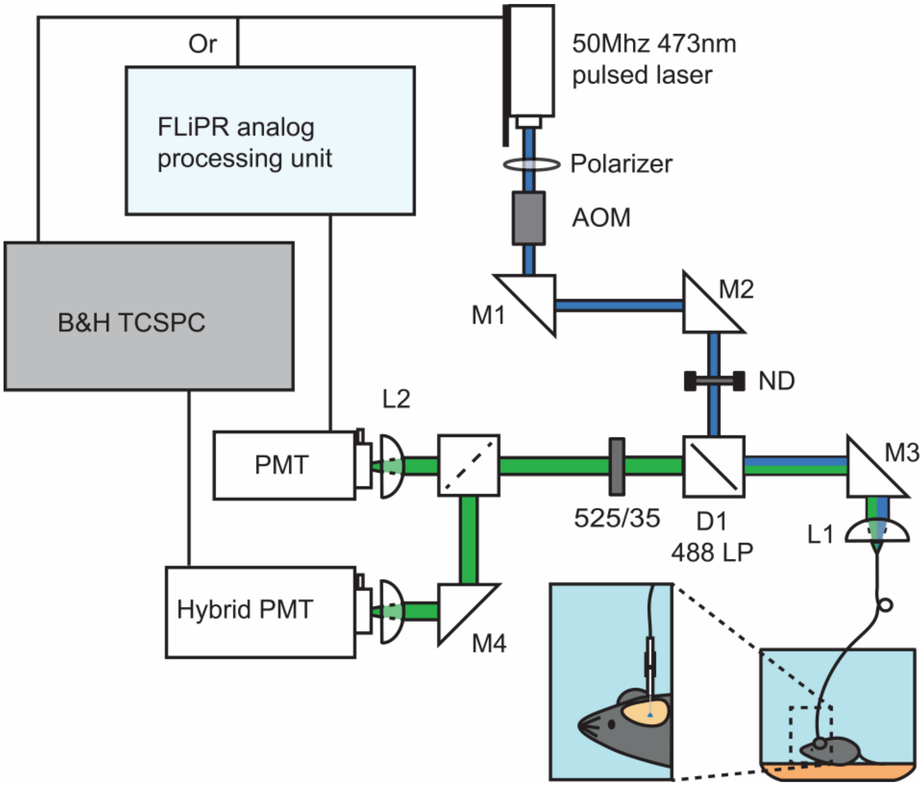

Figure S2. Optical set-up for comparison of FLIP and FLIPR, Related to Figure 1
Detailed diagram of the optical system used for comparison of fluorescence lifetimes measured with time domain photometry (FLiP) and frequency domain FLIPR. AOM: acousto-optic modulator; M#: mirror; ND, neutral density filter; D1: dichroic: L#: lens; PMT, photon multiplier tube; B&H TCSPC: Becker and Hickl time correlated single photon counting board.

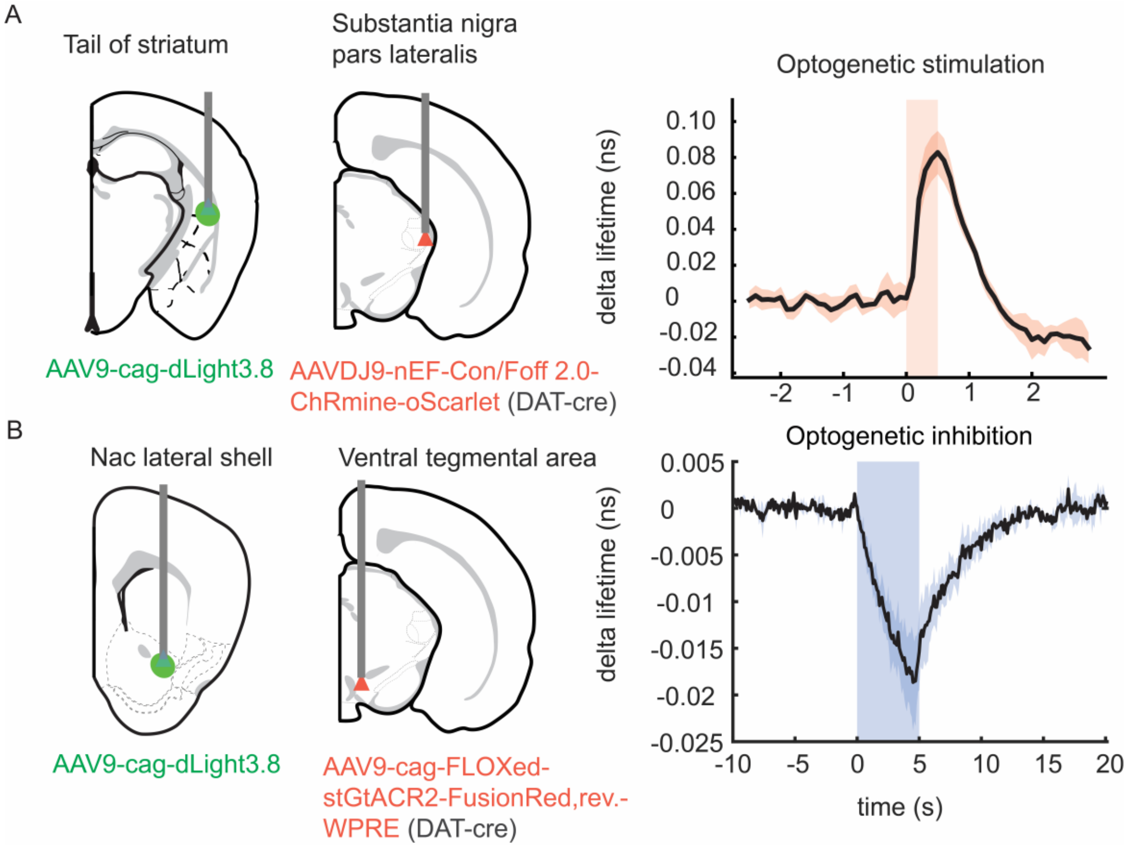

Figure S3. dLight3.8 measurement and manipulation of dopamine neurons, Related to Figure 4

A, Optogenetic stimulation of dopamine neurons increases fluorescence lifetime of dLight3.8 expressed in the tail of striatum (TOS). ChRmine was expressed in dopamine neurons of the substantia nigra pars lateralis (SNPL), which projects to TOS, using Cre-dependent AAV injected into DAT-cre mice (*left*). Fiber optics were placed above the TOS and SNPL for FLIP recording and optogenetic stimulation, respectively. Optogenetic stimulation of SNPL dopamine neurons increased fluorescence lifetime of dLight3.8 in the TOS (*right*).

B, As in A but using stGtACR2 to suppress activity of ventral tegmental area (VTA) dopamine neurons that project to the dLight3.8 measurement site in NAC lateral shell (NACls) (*left*). Optogenetic inhibition (5 s, continuous, 6 mW) of VTA dopamine neurons decreased fluorescence lifetime of dLight3.8 in the NACls.

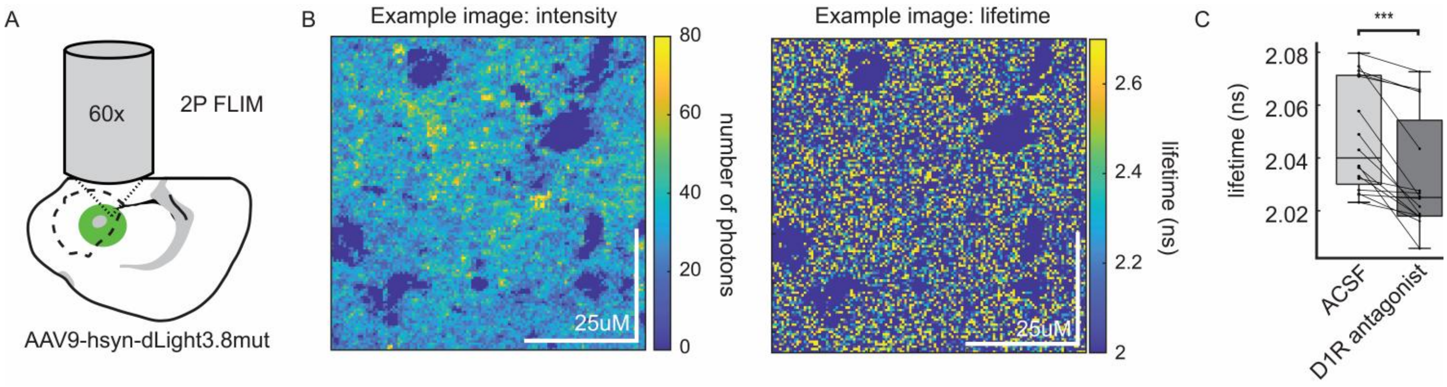

Figure S4. dLight3.8mut response to non-competitive D1R antagonist in brain slice, Related to Figure 4

A, dLight3.8mut expressed in the nucleus accumbens was measured using two-photon fluorescence lifetime microscopy. dLight3.8mut lifetime was measured in ACSF and in ACSF + 10μM of D1R antagonist SCH23390.

B, Intensity (left) and lifetime (right) example image of nucleus accumbens site expressing dLight3.8mut,

C, D1R antagonist SCH23390 significantly decreased fluorescence lifetime of dLight3.8mut (paired sample T-test, p=1.68E-4, n=16 sites across 3 animals)

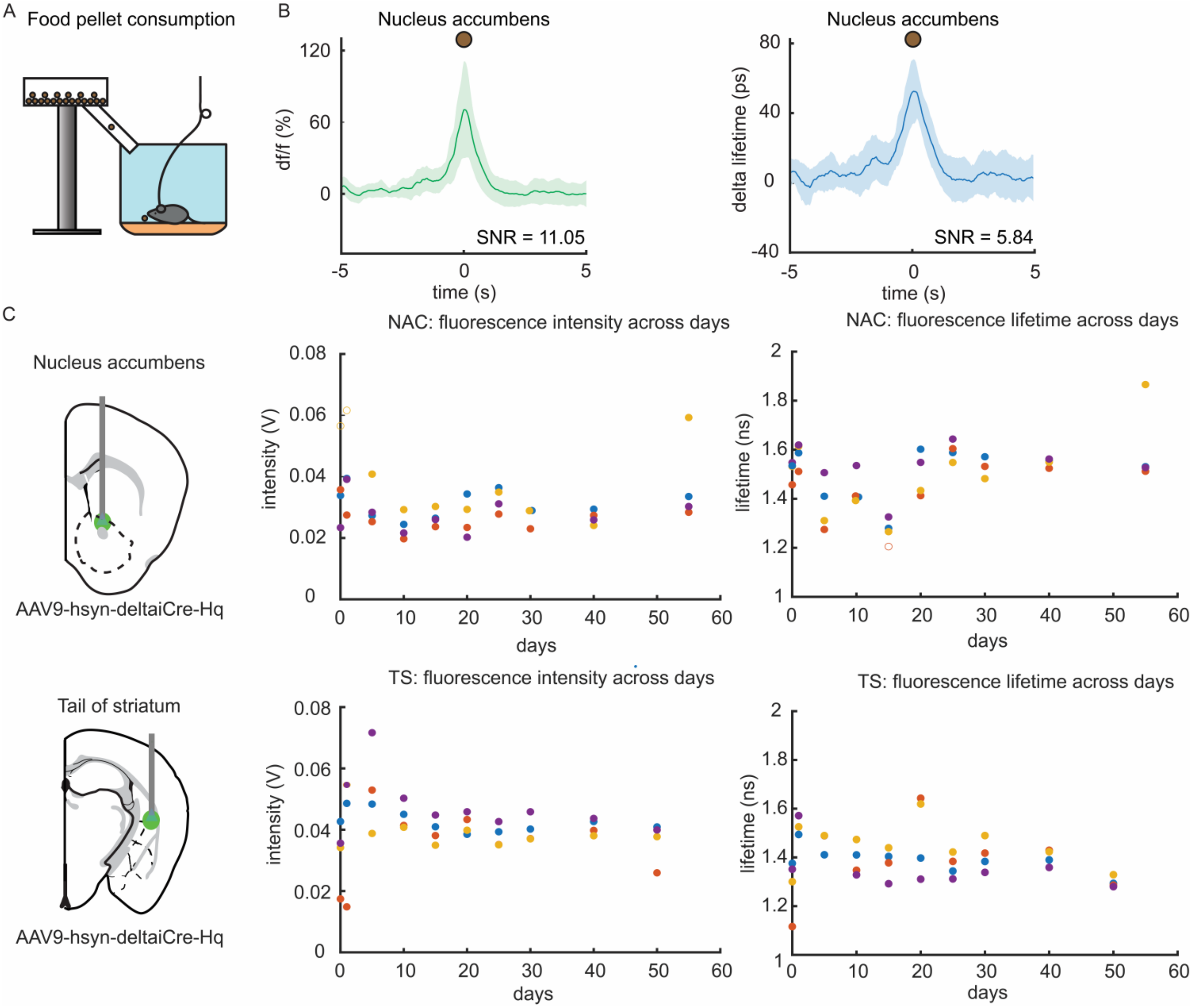

Figure S5. Signal to noise ratio comparison of intensity and lifetime dLight3.8 signal in the accumbens and autofluorescence across days, Related to Figure 5 and 6

A, Schematics of devices for spontaneous delivery of food pellets.

B, intensity and lifetime response of dLight3.8 in the NAC in response to chocolate pellet consumption. The signal to noise ratio (SNR) was greater in the intensity measurement (SNR = 11.05, n = 9 sites) compared to the lifetime measurement (SNR = 5.84, n = 9 sites)

C, Inactivated Cre with no fluorophore tag was expressed in the NAC and TOS, and a fiber optic cannula was placed above the injection site. The lifetime and intensity of the autofluorescence of each brain region were measured using FLIPR across days. Autofluorescence has low brightness compared to typical fluorescent sensor expression and therefore requires very high laser power (>100μW) to reach adequate photon levels for accurate lifetime measurement. The high variability of lifetime and intensity autofluorescence measurement compared to typical sensor measurements are likely explained by variation in patch-cord to implant coupling at high power and subsequent changes in the ratio of autofluorescence contribution of the patch cord compared to brain tissue. During typical sensor measurements, where the brightness of the sensor is considerably higher than autofluorescence, the effect of changes in patch-cord to implant coupling on lifetime measurements are negligible (see also **Figure 3B** and main text). n=4 sites

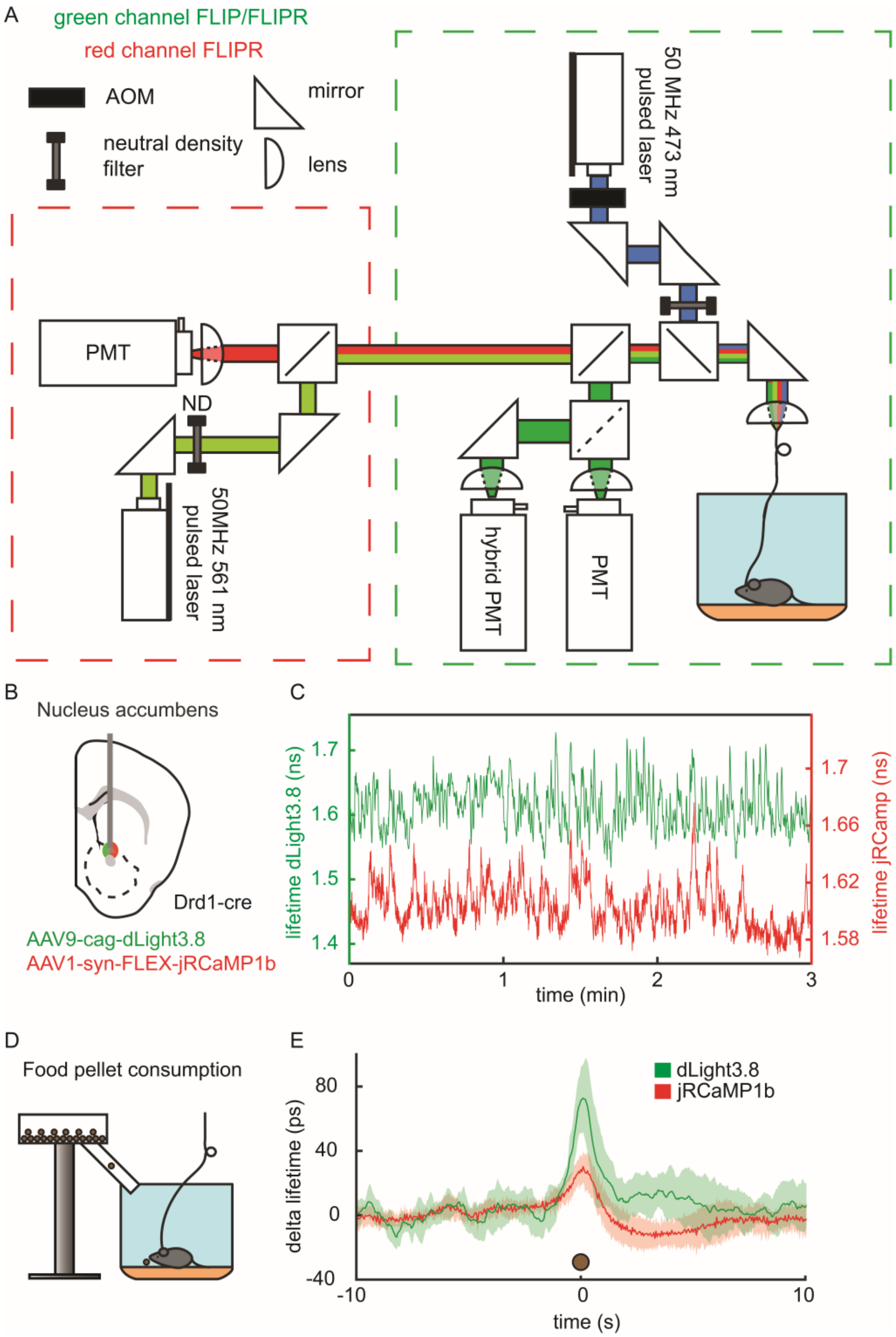

Figure S6. Two-color FLIPR system allows for high-speed measurement of two fluorescence lifetime sensors, Related to Figure 6

A, Schematic of two-color FLIPR including a green and red channel. A red FLIPR channel was added to the green FLIPR system as described in Figure 1, S1-3. A 561 nm 50 MHz pulsed laser was power modulated using a variable neutral density filter and directed into the patchcord. Emission light was separated from the green channel and 561 nm excitation light using dichroic and bandpass filters and captured in a photon multiplier tube (PMT). The 561 nm laser reference pulse and PMT signal were processed in a separate FLIPR system similar to the system described in Figure 1, S1-3.

B, green dopamine sensor dLight3.8 was expressed simultaneously with cre dependent red calcium sensor jRCaMP in the nucleus accumbens of dopamine 1 receptor cre (D1-cre) mice.

C, Example trace of simultaneous two-color measurement of dLight3.8 and jRCaMP.

D, Schematics of devices for spontaneous delivery of food pellets.

E, dLight3.8 (green) and jRCaMP (red) lifetime response to food pellet consumption (n = 7 sites).
